# Spatial multi-omics analysis of tumor-stroma boundary cell features for predicting breast cancer progression and therapy response

**DOI:** 10.3389/fcell.2025.1570696

**Published:** 2025-03-26

**Authors:** Yuanyuan Wu, Youyang Shi, Zhanyang Luo, Xiqiu Zhou, Yonghao Chen, Xiaoyun Song, Sheng Liu

**Affiliations:** ^1^ Department of Breast Surgery, Longhua Hospital, Shanghai University of Traditional Chinese Medicine, Shanghai, China; ^2^ Shanghai Pudong Hospital, Fudan University Pudong Medical Center, Shanghai, China; ^3^ West China Hospital of Sichuan University, Chengdu, China

**Keywords:** breast cancer, spatial transcriptomics, tumor boundary, CAF-M2 interaction, therapy resistance, prognostic model

## Abstract

**Background:**

The tumor boundary of breast cancer represents a highly heterogeneous region. In this area, the interactions between malignant and non-malignant cells influence tumor progression, immune evasion, and drug resistance. However, the spatial transcriptional profile of the tumor boundary and its role in the prognosis and treatment response of breast cancer remain unclear.

**Method:**

Utilizing the Cottrazm algorithm, we reconstructed the intricate boundaries and identified differentially expressed genes (DEGs) associated with these regions. Cell-cell co-positioning analysis was conducted using SpaCET, which revealed key interactions between tumor-associated macrophage (TAMs) and cancer-associated fibroblasts (CAFs). Additionally, Lasso regression analysis was employed to develop a malignant body signature (MBS), which was subsequently validated using the TCGA dataset for prognosis prediction and treatment response assessment.

**Results:**

Our research indicates that the tumor boundary is characterized by a rich reconstruction of the extracellular matrix (ECM), immunomodulatory regulation, and the epithelial-to-mesenchymal transition (EMT), underscoring its significance in tumor progression. Spatial colocalization analysis reveals a significant interaction between CAFs and M2-like tumor-associated macrophage (TAM), which contributes to immune exclusion and drug resistance. The MBS score effectively stratifies patients into high-risk groups, with survival outcomes for patients exhibiting high MBS scores being significantly poorer. Furthermore, drug sensitivity analysis demonstrates that high-MB tumors had poor response to chemotherapy strategies, highlighting the role of the tumor boundary in modulating therapeutic efficacy.

**Conclusion:**

Collectively, we investigate the spatial transcription group and bulk data to elucidate the characteristics of tumor boundary molecules in breast cancer. The CAF-M2 phenotype emerges as a critical determinant of immunosuppression and drug resistance, suggesting that targeting this interaction may improve treatment responses. Furthermore, the MBS serves as a novel prognostic tool and offers potential strategies for guiding personalized treatment approaches in breast cancer.

## Introduction

Breast cancer (BRCA) is the most prevalent malignancy among women and the second leading cause of cancer-related mortality in this population. In 2023, it was estimated that 43,170 women in the United States died from breast cancer (Nicholson et al., 2024; Liang et al., 2020). The annual incidence rate of newly diagnosed breast cancer cases in women was 129.4 per 100,000 individuals. Between 2017 and 2021, the mortality rate for breast cancer was 19.3 per 100,000 women annually (National Cancer Institute, 2025). Over the past few decades, clinical outcomes for breast cancer patients have progressively improved, owing to the widespread implementation of screening programs and advances in therapeutic strategies (Qi et al., 2024). Both public and private investments in research, as well as the translation of research findings into clinical practice, have contributed to a substantial decline in breast cancer mortality over the past five decades (Wheeler et al., 2024). However, despite significant progress in early detection and treatment, many patients still experience disease progression and suboptimal therapeutic outcomes. A deeper understanding of the molecular and cellular mechanisms underlying breast cancer progression, as well as factors influencing therapeutic response, is critical for further improving patient outcomes (Khan et al., 2024; Ye et al., 2023).

Recent studies have highlighted the crucial role of the tumor microenvironment (TME) in both breast cancer progression and therapeutic responses. The TME is a complex and dynamic ecosystem composed of various cell types, including cancer cells, stromal cells, immune cells, and vascular cells (Xiao and Yu, 2021; Elhanani et al., 2023). The interactions among these cellular components are essential for tumor growth, invasion, and response to treatment. Notably, the tumor-stroma interface serves as a dynamic boundary where cancer cells and stromal cells engage in intricate interactions. The cellular characteristics at this interface provide valuable insights into the mechanisms driving disease progression and may serve as predictive markers for therapeutic responses (Bader et al., 2020; Tang et al., 2021; Toninelli et al., 2023).

As our understanding of tumor biology advances, the importance of spatial context—encompassing both cell localization and the interactions among various components of the tumor microenvironment—has become increasingly apparent. This growing recognition has led to the development of spatial multi-omics, a comprehensive approach for examining the intricate architecture of the tumor microenvironment. By focusing on the spatial organization of cellular and molecular profiles both within and between different tumor compartments, spatial multi-omics provides unprecedented insights into tumor biology. In this review, we will explore the applications of spatial multi-omics in identifying therapeutic targets, predicting treatment responses, and advancing precision medicine, while also addressing the challenges and future directions of this emerging field (Du et al., 2024; Hsieh et al., 2022; Wu et al., 2022).

Spatial multi-omics analysis is an emerging technique that integrates multiple layers of molecular and cellular data to provide a comprehensive characterization of the tumor ecosystem, enabling the concurrent examination of DNA, RNA, protein, and metabolite profiles in a spatially resolved context, which facilitates the identification of distinct cellular phenotypes and their interactions within the tumor microenvironment. By focusing on the tumor-stroma interface, spatial multi-omics analysis offers the potential to reveal unique cellular features that are associated with breast cancer progression and therapeutic responses.

Previous studies have identified various clinical, molecular, and digital pathology features associated with responses to neoadjuvant therapy in breast cancer (Jin et al., 2023). However, these studies often relied on single-platform profiling, which failed to capture the full complexity of the tumor ecosystem (Liang et al., 2020). More recent research has demonstrated that integrating multi-omic data—encompassing clinicopathological variables, digital pathology, and both DNA and RNA sequencing—can significantly improve the accuracy of predictive models for therapeutic responses. For example, one study showed that combining clinical, genomic, and transcriptomic features with digital pathology data resulted in robust predictive models for pathologic complete response (pCR) in breast cancer patients undergoing neoadjuvant therapy (Nolan et al., 2023; Sammut et al., 2022).

In this study, we aim to perform a spatial multi-omics analysis of the cellular features at the tumor-stroma boundary to predict breast cancer progression and therapeutic responses. By integrating multi-omic data from this critical interface, we seek to identify novel cellular and molecular markers that could improve the accuracy of predictive models, while providing deeper insights into the underlying mechanisms of breast cancer progression and therapy resistance. This approach holds significant potential for enhancing clinical decision-making and advancing personalized treatment strategies for breast cancer patients.

## Materials and methods

### Spatial transcriptome data collection and preprocessing

Spatial transcriptome (ST) data of breast cancer was retrieved from GEO database (https://www.ncbi.nlm.nih.gov/geo/) and 10x Genomics official website (https://www.10xgenomics.com/). Firstly, we screened the 59-breast cancer spatial transcriptomics samples provided by the SpatialTME database (accessible at https://www.spatialtme.yelab.site/) and identified eight samples with typical tumor boundary structures. Subsequently, we selected four representative samples from these for further analysis. Among the four samples, two are from the 10x platform: 10x BRCA (ductal carcinoma *in situ* and invasive carcinoma) and 10x BRCA2 (Group IIA, ER+, PR-, Her2+, diagnosed with ductal carcinoma *in situ*, invasive carcinoma, and lobular carcinoma *in situ*). Additionally, we included GSM6433610 from the GSE210616 dataset (derived from triple-negative breast cancer) (Bassiouni et al., 2023), and GSM6177603 from the GSE203612 dataset (derived from invasive lobular carcinoma) (Barkley et al., 2022). All four samples are 10x Genomics data. The ST data was processed and analyzed using the Seurat R software package. To achieve standardization of the ST data, the SCTransform (SCT) method was employed, which involved functions such as SelectIntegrationFeatures, PrepSCTIntegration, FindIntegrationAnchors, and IntegrateData to consolidate the ST datasets. To standardize sparse, count-based data, utilize “Spatial” analysis alongside the “poisson” method. Following standardization, merge the data and designate the DefaultAssay as “SCT”. Perform principal component analysis (PCA) on “SCT” for dimensionality reduction, and apply the Louvain algorithm for clustering with a resolution set to 0.6. The identification of cell populations was informed by hematoxylin and eosin-stained (HE) sections, along with the detection of significantly variable genes in each cluster. To visualize the expression levels of cells within the ST data, the functions SpatialDimPlot and SpatialFeaturePlot were effectively used together.

### Reconstruction of malignant-boundary axis

The tumor border, which encompasses both malignant and non-malignant cells, represents a highly heterogeneous region characterized by interactions between cancer cells and various cell types, including immune and stromal cells. We defined the tumor boundary using the Cottrazm package (Xun et al., 2023), categorizing it into three areas: the malignant (Mal) area, the tumor boundary (Bdy), and the non-malignant (nMal) area. Additionally, we calculated the differential genes associated with Bdy to further characterize the properties of the tumor boundary.

### Identification of upregulated genes of Bdy region

By applying a predetermined threshold of p < 0.05 and log2fc > 0.25, we identified differentially expressed genes (DEGs) between the Bdy and other regions. Additionally, the “clusterProfiler 4.0” R package was employed to explore signaling pathways linked with the DEGs, annotated according to Gene Ontology (GO) and Kyoto Encyclopedia of Genes and Genomes (KEGG) and HALLMARKE (Wu et al., 2021).

### Cell to cell colocalization and correlation analysis

Due to the unique nature of the Space Transcription Group (ST), each spatial point (SPOT) encompasses multiple cell signals. We utilize SpaCET (V1.0.0) software to identify the cell types present within the cells included in the ST data set (Ru et al., 2023). After analyzing cellular components, SpaCET can infer cell-cell interactions based on cellular co-localization and ligand-receptor co-expression. Linear correlations of cell fractions were calculated across all ST points to assess the co-localization of cell types. The functions SpaCET.CCI.colocalization and SpaCET.visualize.colocalization were utilized to calculate and visualize the pairs of colocalized cell types.

### Bulk data collection and processing

Data regarding gene expression and comprehensive clinical information for BRCA patients were sourced from The Cancer Genome Atlas (TCGA, https://portal.gdc.cancer.gov/). In total, 1,055 samples were ultimately included in the analysis. The gene sequencing results across the three cohorts were represented in transcripts per million (TPM) formats, and the expression data underwent a pre-transformation to log2 (TPM +1) to ensure comparability. Noise was characterized as mRNAs with a TPM value of less than 1 present in over 90% of the samples, which were then excluded. Patients lacking paired mRNA profiles or clinical data, as well as those without follow-up information, were removed to reduce potential biases. The outcome variable was defined as overall survival (OS).

### Construction of Malignant-boundary prognostic model

Using the “GLMNET” package, we performed a regression analysis that utilized the Least Absolute Shrinkage and Selection Operator (LASSO) to identify the ultimate variables and compute the necessary coefficients for creating the Malignant-boundary Signature (MBS). Typically, the optimal value of lambda is established through cross-validation methods, including techniques like 10-fold cross-validation, to find an ideal equilibrium between bias and variance, which in turn improves the predictive accuracy of the model. In our research, a lambda value of 0.008 was obtained through cross-validation, offering the best compromise between reducing model complexity and ensuring high predictive precision. The risk score was then calculated using the following formula:

$$MBS\;score=\sum_{k=1}^{n}\left(coef.i*expression.i\right)$$

### Chemotherapeutic response evaluation

Concerning the initial chemotherapy protocol for BRCA and the acknowledged activation of signaling pathways, we selected specific drugs to evaluate the predictive therapeutic potential of our model. Relevant data were gathered from GDSC 2016 (https://www.cancerrxgene.org/) and subsequently integrated into the ComDrug program within the “MOVICS” package (Lu et al., 2020). For each patient, we applied ridge regression analysis to determine the estimated inhibitory concentration (IC50), which indicates their responses to various medications.

### TIDE (tumor immune dysfunction and exclusion) analysis

To evaluate immune dysfunction and immune exclusion within the tumor microenvironment, we employed the TIDE model. The TIDE score predicts tumor response to immunotherapy by analyzing mechanisms of immune evasion in tumors, including immune checkpoint inhibition. This model assesses two critical factors: tumor immune dysfunction and immune exclusion. Based on these two factors, the TIDE score is calculated, with higher scores indicating more severe immune dysfunction or exclusion and a poorer response to immune checkpoint inhibitors. Patients were stratified into responder and non-responder groups according to their TIDE scores, and a chi-square test was used.

### Multiplex immunofluorescence (mIF) analysis

A multiplex immunofluorescence (mIF) technique was utilized to assess the spatial arrangement and co-expression of targeted markers within the tumor microenvironment. Tissue sections that were fixed in formalin and embedded in paraffin (FFPE) underwent a series of staining procedures using the Opal™ multiplex immunofluorescence system (Akoya Biosciences). The primary antibodies deployed included CD68, CD163, FAP, CD8, and ACTA2. For nuclear counterstaining, DAPI (4′, 6-diamidino-2-phenylindole) was utilized. Following deparaffinization and rehydration, an antigen retrieval process was conducted using a Tris-EDTA buffer at a pH of 9.0, employing a pressure cooker. To minimize non-specific binding, the tissue sections were treated with a protein blocking buffer. Each primary antibody was added in succession, followed by the relevant Opal fluorophore-conjugated secondary antibody. After each round of staining, the antigen retrieval was repeated to remove the preceding antibody complex without compromising the fluorophore conjugates (Xu et al., 2023; Xu et al., 2020). The fluorophores employed in the study included Opal 620 designated for CD68, Opal 570 for CD163, Opal 620 for CD8, Opal 690 for ACTA2, and Opal 520 for FAP. Additionally, DAPI was included for the purpose of visualizing the nuclei.

### Statistical analysis

Outcomes related to survival were assessed using the log-rank test, while the analysis of categorical data utilized Fisher’s exact test. The comparison between the high-MBS and low-MBS subgroups was conducted through Student’s t-test. All statistical evaluations were carried out using R (Version: 4.2.1). A two-tailed p-value of less than 0.05 was considered statistically significant.

## Results

### The tumor boundary contains heterogeneous cells and signals

**FIGURE 1:**
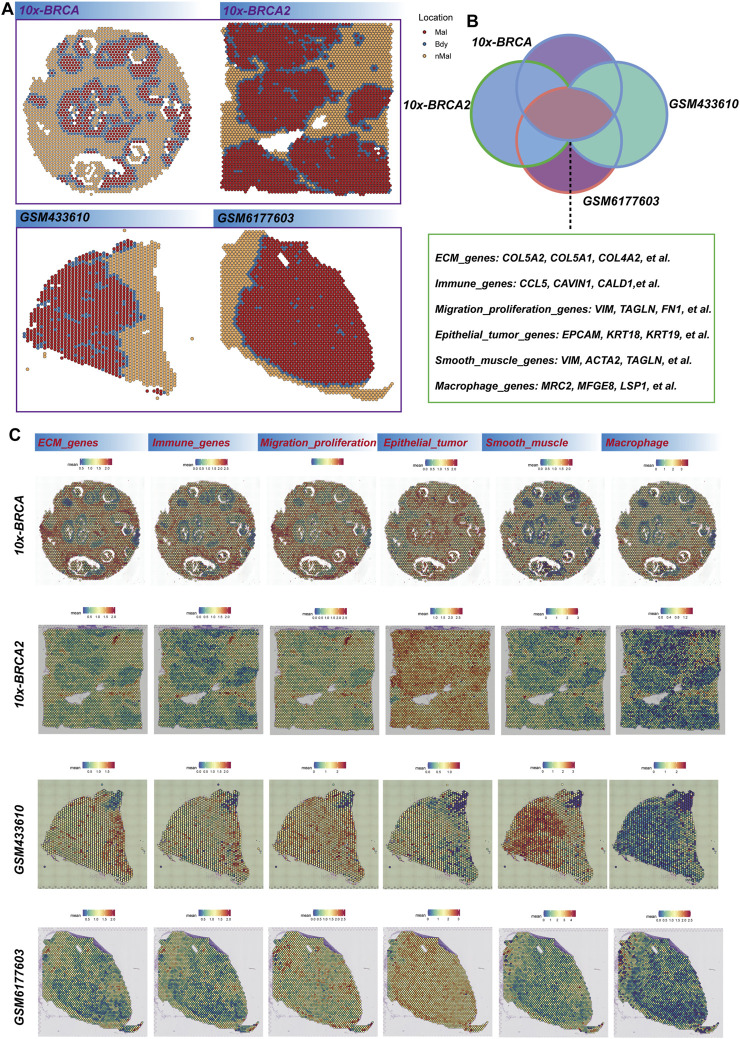
Breast cancer tumor boundary reconstruction. **(A)** The Cottrazm algorithm is employed to determine the spatial distribution of malignant (Mal), boundary (Bdy), and non-malignant (nMal) regions across four breast cancer datasets. **(B)** Venn diagram illustrates the overlap of differentially expressed genes (DEGs) among these datasets. The genes are categorized into primary functional groups, including extracellular matrix (ECM) genes (e.g., COL5A2, COL5A1, COL4A2), immune-related genes (e.g., CCL5, CAVIN1, CALD1), migration and proliferation genes (e.g., VIM, TAGLN, FN1), epithelial tumor markers (e.g., EPCAM, KRT18, KRT19), smooth muscle-related genes (e.g., VIM, ACTA2, TAGLN), and macrophage-related genes (e.g., MRC2, MFGE8, LSP1). **(C)** Spatial scores of key gene characteristics in breast cancer samples are presented, with each row representing different breast cancer samples (10x-BRCA, 10X-BRCA2, GSM433610, GSM6177603) and each column corresponding to specific gene categories.

We employ the Cottrazm algorithm to reconstruct the tumor boundary, differentiating between tumor tissue and non-tumor tissue in our analysis of the spatial transcription group (Figure 1A). High-expression genes of the four samples are extracted from each sample’s tumor boundary (p < 0.05 and log2fc > 0.25) (Supplementary Table S1). The intersection of highly expressed genes was identified, and a total of 55 shared genes were confirmed (Supplementary Table S2). These genes predominantly encompass extracellular matrix (ECM) related genes, immune-related genes, migration and proliferation-related genes, epithelial-related genes, smooth muscle-related genes, and macrophage-related genes (Figure 1B). Subsequently, we visualize the spatial expression patterns of these genes across various datasets, including 10X-BRCA, 10X-BRCA2, and additional samples such as GSM433610 and GSM6177603. The spatial diagrams illustrate the differential expression of these genes in comparison to tumor and non-tumor regions within the tumor boundary. Notably, ECM genes (e.g., Col5A2 and COL5A1) and immune-related genes (e.g., CCL5 and CAV1) exhibit significant expression patterns, suggesting the presence of a dynamic immune environment. The tumor boundary is enriched with migration-related genes such as VIM and FNT, indicating active tumor cell migration and proliferation. Furthermore, epithelial markers (e.g., EPCAM and KRT18), smooth muscle-related genes (e.g., ACTA2), and macrophage markers (e.g., MRC2) are distinctly expressed in the border area, providing valuable insights into the tumor microenvironment (Figure 1C). These findings offer a comprehensive perspective on the spatial heterogeneity of breast cancer tissue and suggest potential therapeutic targets associated with the tumor boundary and its surrounding microenvironment.

### Progression-related pathway activation along with tumor boundary

**FIGURE 2:**
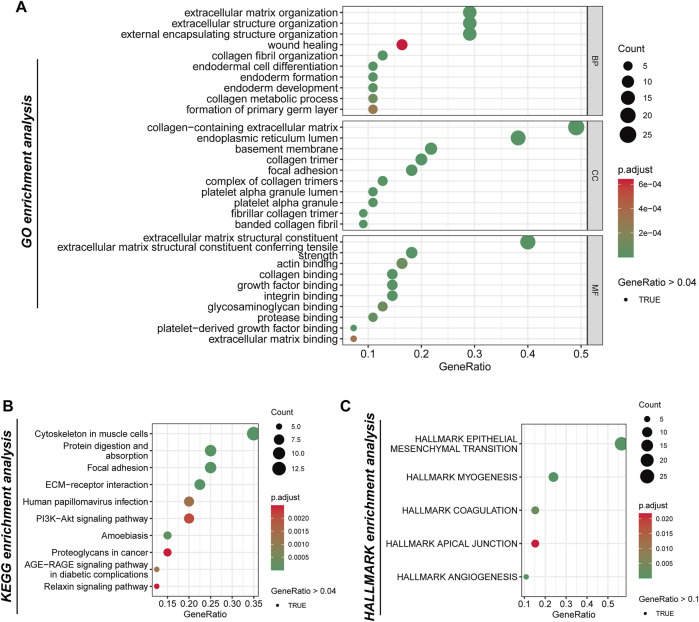
The analysis of the genetic functions of tumor boundary high-expression genes. **(A)** Gene Ontology (GO) enrichment analysis categorizes the border-related genes into three primary functional groups: biological processes (BP), cellular components (CC), and molecular functions (MF). **(B)** The analysis of the Kyoto Encyclopedia of Genes and Genomes (KEGG) pathways reveals significant enrichment within specific pathways. **(C)** Examination of the Hallmark gene collection identifies five principal pathways.

In the analysis of boundary gene richness, we conducted a genetic analysis using Gene Ontology (GO), KEGG, and Hallmark pathways. Our findings highlight the significance of tumor boundaries in key processes, including the extracellular matrix (ECM), cell migration, immune regulation, and tumor progression. GO enrichment analysis revealed several significant categories related to the ECM and cellular structure. The most notably enriched terms pertained to ECM organization and wound healing, underscoring the critical role that border genes play in the dynamic remodeling of the ECM, which is essential for tumor progression and metastasis (Figure 2A). Additionally, genes associated with collagen fiber organization, basement membrane formation, and focal adhesions were also enriched, indicating that the tumor border contributes to maintaining the structural integrity of the tissue while facilitating interactions between tumor cells and the surrounding matrix. Other enriched GO terms, such as endoderm cell differentiation, endoderm formation, and collagen-containing extracellular matrix, further emphasize the involvement of boundary genes in cell differentiation and tissue development processes that may influence tumor heterogeneity and progression. KEGG pathway enrichment analysis further elucidates the role of ECM remodeling in tumor progression, highlighting significant enrichment in ECM-receptor interaction pathways. This underscores the importance of interactions between ECM components and tumor cells. Additionally, pathways related to the cytoskeleton and protein digestion and absorption were enriched in muscle cells, indicating that the mechanical properties and flexibility of tumor cells at their borders are crucial for their migration and invasion into adjacent tissues. Furthermore, the enrichment of the PI3K-Akt signaling pathway, which is vital for cell survival, proliferation, and metabolism, suggests that border genes may regulate key signaling pathways that facilitate tumorigenesis and confer resistance to treatment. The significant enrichment of the human papillomavirus infection pathway indicates a potential influence of the virus on the development or progression of specific tumor types, although further research is necessary to clarify its relevance in this context (Figure 2B). Moreover, the enrichment of the AGE-RAGE signaling pathway, known to be associated with inflammation, fibrosis, and cancer, further implies that border genes may contribute to the inflammatory microenvironment of tumors. HALLMARK pathway enrichment analysis identified several key pathways involved in tumor progression. The most significantly enriched pathway was the epithelial-to-mesenchymal transition (EMT), a process recognized as critical for cancer cell metastasis. The enrichment of this pathway suggests that border genes may facilitate the transition from an epithelial to a mesenchymal phenotype, thereby enhancing cell migration and invasion (Figure 2C). Additionally, myogenic pathways related to myocyte differentiation were also enriched, indicating the potential influence of the tumor microenvironment on the differentiation and proliferation of surrounding stromal cells. Apical junctional pathways were highlighted, underscoring the importance of intercellular adhesion and junctional integrity in maintaining tissue structure, which may also affect the ability of tumors to invade adjacent tissues. Finally, angiogenesis, the formation of new blood vessels, was significantly enriched, further supporting the notion that border genes contribute to promoting tumor vascularization, a process essential for tumor growth and metastasis.

### Presence of Cancer-associated fibroblasts (CAF)-M2 tumor-associated macrophage (TAM) structure in the periphery of breast cancer

**FIGURE 3:**
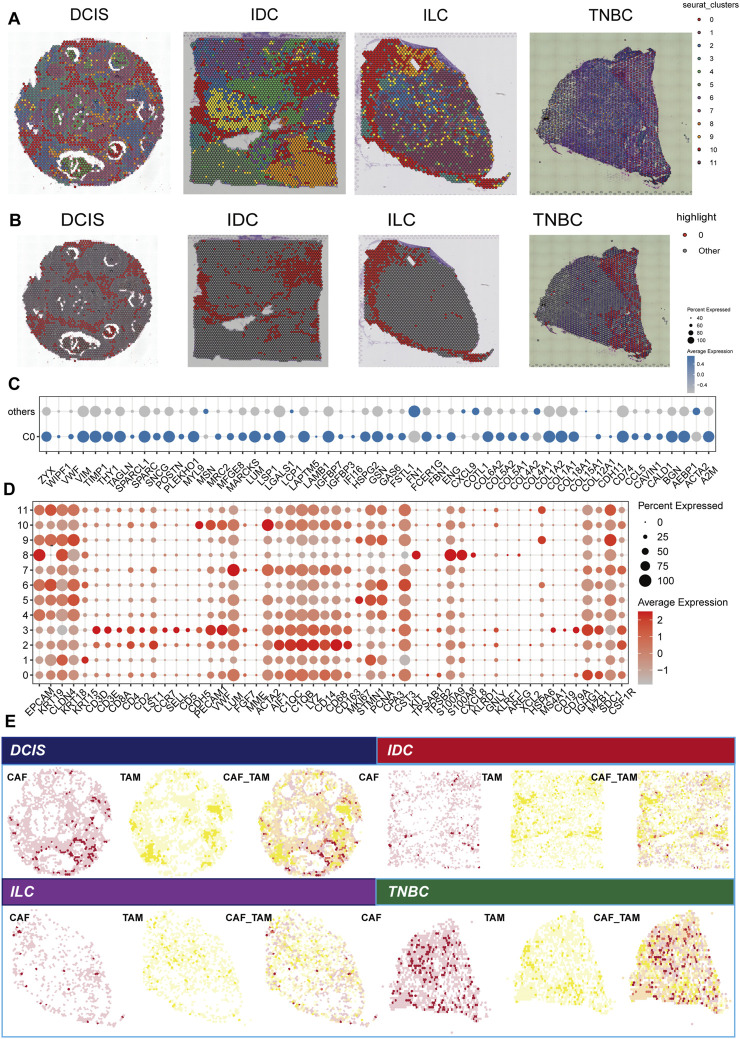
Analysis of spatial transcription groups across various breast cancer subtypes. **(A)** Spatial clustering of breast cancer tissue samples, including ductal carcinoma *in situ* (DCIS), infiltrating ductal carcinoma (IDC), infiltrating lobular carcinoma (ILC), and triple-negative breast cancer (TNBC). Each point represents a spatial transcription group. **(B)** The spatial distribution of the C0 cluster. **(C)** Comparison of boundary gene expression between cluster C0 and other clusters. Each point represents a gene, indicating the proportion of the gene’s presence, with color denoting the average expression level. **(D)** DEGs among 12 clusters identified in the spatial transcription group data. Point size reflects the proportion of points corresponding to a given gene, while color intensity represents the level of gene expression. **(E)** Spatial localization of cancer-associated fibroblasts (CAFs) and tumor-associated macrophage (TAMs). The left panel for each subtype presented the CAF score distribution, the middle panel shows the TAM score distribution, and the right panel illustrates the combined positioning (CAF_TAM) of CAF and TAM scores, highlighting potential interactions within the tumor microenvironment.

In this analysis, we utilized the Seurat to cluster spatial transcriptome data from four distinct breast cancer subtypes: DCIS, IDC, ILC, and TNBC. The clustering algorithm identified a total of 12 clusters (Figure 3A), with cluster 0 representing tumor boundary tissue (Figure 3B). Through differential gene expression analysis, combined with morphological characteristics and the previously defined tumor boundary, we determined that subcluster 0 is the primary component of tumor boundary tissue. Furthermore, we compared the differences in boundary gene expression between C0 and the other clusters. The boundary genes in C0 exhibited generally high expression levels in this region, further confirming its designation as a border area (Figure 3C). Difference analysis revealed that subcluster 0 has elevated expression of markers such as EPCAM, KRT19, PECAM1, ACTA2, C1QB, and CD79A, indicating that it comprises a mixture of epithelium, endothelium, fibroblasts, macrophages, and B cells (Figure 3D). Notably, the high expression of CD163 and CD68 suggests a predominant presence of M2 macrophages. Previous studies by Wu et al. (2023) defined an invasion area characterized by a high infiltration of M2 cells, contributing to a relatively suppressive immune microenvironment that facilitates the progression of liver cancer. In our study, we similarly found that the breast cancer border is predominantly composed of M2 cells. Consequently, we further calculated the scores of cancer-associated fibroblasts (CAF) and M2 tumor-associated macrophages (TAM), and spatially visualized their co-localization, confirming the existence of CAF-M2 TAM in the border tissue of breast cancer (Figure 3E).

### The co-localization of FAP^+^ACTA2^+^CAF and CD68^+^CD163^+^M2 like TAM at the tumor boundary may be a key factor driving breast cancer progression

**FIGURE 4:**
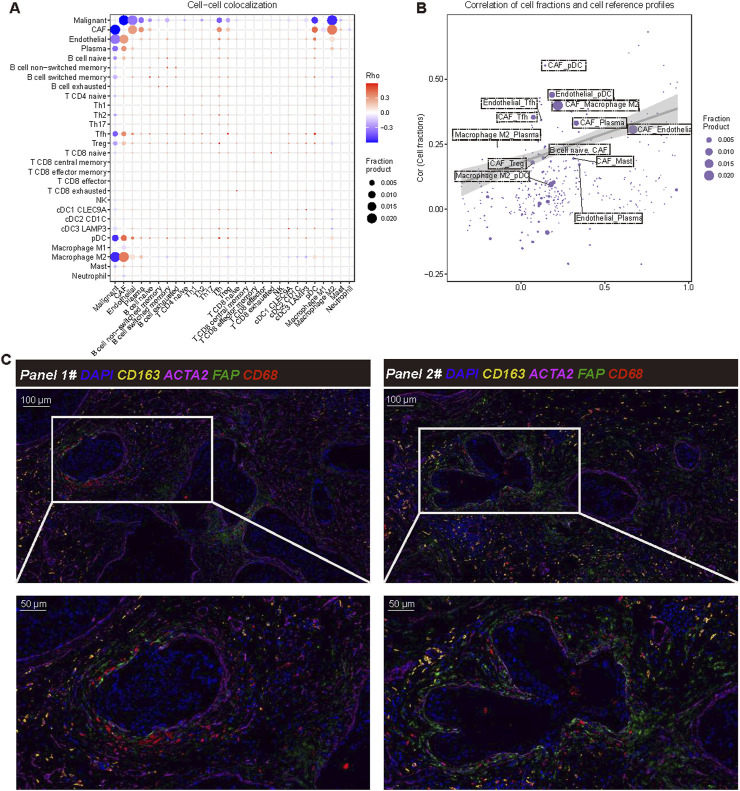
Cell-cell colocalization analysis and interaction analysis. **(A)** Cell to cell colocalization analysis using SpaCET illustrates the spatial relationships among different cell types within the tumor microenvironment. Each point represents a pairwise co-localization of these cell types, with the Rho value (Spearman’s rank correlation coefficient) indicating the strength of the co-localization. Larger dots correspond to higher fraction products, indicating more frequent colocalization between cell types. The color scale represents Rho values, where red and blue indicate positive and negative correlations, respectively. Statistical significance of co-localizations was assessed using Spearman’s correlation. **(B)** The correlation between cell scores and reference cells is depicted, where each point represents a specific cell type. This representation shows the correlation between estimated cell scores (Y-axis) and reference profiles (X-axis). The size of each point corresponds to the score product, and the shaded area represents the confidence range of the model’s output. **(C)** Verification through multiple immunofluorescence staining demonstrates the co-localization of cancer-associated fibroblasts (CAF) and macrophages within the tumor microenvironment. The markers used include DAPI (nucleus, blue), ACTA2 (CAF, purple), FAP (fibroblast activation protein, green), CD68 (all macrophages, yellow), and CD163 (M2 macrophage marker, orange). The magnified area highlights the spatial interaction between CAF and M2 macrophages.

Furthermore, we employed the SpaCET deconvolution method to investigate the spatial positioning of various cell types within the tumor microenvironment. The cell-cell colocalization analysis the interactions among different cell types (Figure 4A). Notably, CAFs, M2 macrophages, and malignant tumor cells exhibit the most pronounced co-localization, underscoring their significant roles at the tumor boundary. These results suggest that CAFs and M2 macrophages may collaborate to facilitate tumor progression, which aligns with previous findings. The correlation diagram of cell components and reference profiles further elucidates the relationships among these cell types. Our analysis reveals strong correlations between CAFs and M2 macrophages, indicating their potential involvement in reshaping and regulating the immune landscape of the tumor microenvironment (Figure 4B). The colocalization of CAFs and M2 macrophages is a critical characteristic of the tumor boundary, suggesting that this arrangement may promote immune evasion and support tumor growth and metastasis. The mIF analysis (Figure 4C) provides visual confirmation of these findings. FAP^+^ACTA2^+^CAF and CD68^+^CD163^+^M2 like TAM were frequently co-localized, particularly in the tumor boundary, thereby reinforcing the spatial correlation between FAP^+^ACTA2^+^CAF and CD68^+^CD163^+^M2 like TAM in the TME.

In the other four independent samples, we also observed the colocalization of FAP^+^ACTA2^+^CAF and CD68^+^CD163^+^M2 like TAM in the tumor boundary (Supplementary Figure S1). These results underscore the significance of the FAP^+^ACTA2^+^CAF and CD68^+^CD163^+^M2 like TAM interaction at the tumor boundary, suggesting their potential role in tumorigenesis, which may contribute to immunosuppression, tissue remodeling, and tumor progression. This highlights the relevance of these interactions as potential therapeutic targets for breast cancer. Furthermore, we focused on the spatial relationship between CD8^+^ T cells, FAP^+^ACTA2^+^CAF, and CD68^+^CD163^+^ M2-like TAM. To this end, we utilized immunofluorescence staining for analysis. The results revealed that FAP^+^ACTA2^+^CAF and CD68^+^CD163^+^ M2-like TAM were closely clustered around the tumor boundary, while CD8^+^ T cells were located outside the tumor immune barrier formed by FAP^+^ACTA2^+^CAF and CD68^+^CD163^+^ M2-like TAM (Supplementary Figure S2). This finding suggests that tumors with this structure may be associated with a locally suppressive microenvironment, potentially leading to tumor progression and immune escape.

### Prognostic model based on Malignant boundary related genes

**FIGURE 5:**
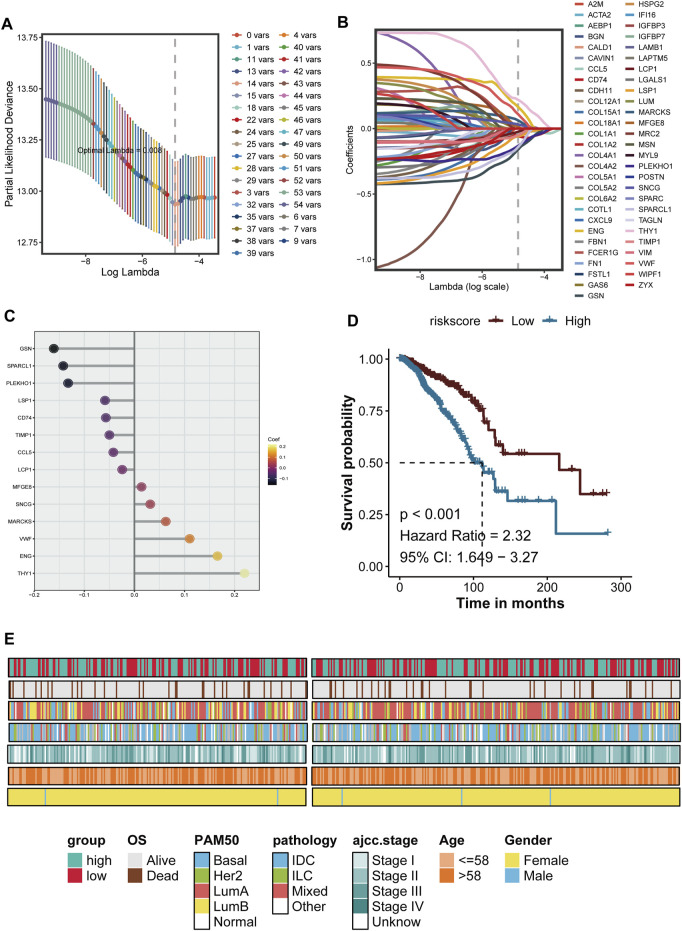
The development and verification of breast cancer prognosis is presented herein. **(A)** The Lasso (Least Absolute Shrinkage and Selection Operator) regression model is utilized for feature selection. The figure illustrates the relationship between the LAMBDA value and the number conversion, with the vertical dotted line indicating the optimal LAMBDA value determined through 10-fold cross-validation, corresponding to the minimum deviation. **(B)** The Lasso coefficient curve of the candidate prognostic genes is depicted, where each colored line represents a gene; the vertical dotted line denotes the selected LAMBDA value retained in the final model. **(C)** The genes and their coefficients used to build the model are showed. **(D)** Kaplan-Meier survival analysis is conducted based on the characteristics of the prognostic genes, comparing high-risk and low-risk groups using a median threshold value. **(E)** The clinical characteristics of patients within the risk groups are summarized. The heat map illustrates the overall survival period (OS), PAM50 molecular subtype, pathology, AJCC staging, age, and gender distribution among high-risk and low-risk groups, indicating a correlation between risk scores and clinical pathological characteristics.

Based on the differential genes identified at the tumor boundary, we utilized Lasso regression to develop a prognostic model. The optimal model, selected from 39 variables exhibiting the lowest likelihood deviation (Figure 5A), allowed us to identify key genes involved in extracellular matrix remodeling, inflammation, and immune response, including A2M, ACTA2, FAP, and HSPG2, and the malignant boundary related signature score = 0.1104 * VWF - 0.0499 * TIMP1 + 0.2200 * THY1 - 0.1422 * SPARCL1 + 0.0317 * SNCG - 0.1324 * PLEKHO1 + 0.0143 * MFGE8 + 0.0626 * MARCKS - 0.0587 * LSP1 - 0.0244 * LCP1 - 0.1609 * GSN +0.1659 * ENG - 0.0570 * CD74–0.0421 * CCL5 (Figures 5B, C). Survival analysis conducted using the Kaplan-Meier method indicates that patients with higher risk scores have a significantly lower survival rate compared to those with lower risk scores (*P* < 0.001), with a risk ratio of 2.32 (95% CI: 1.649–3.27) (Figure 5D). By correlating risk scores with clinical characteristics such as PAM50 subtypes, AJCC stages, age, and gender, we further validated the prognostic accuracy of the model. High-risk patients predominantly belong to advanced cancer stages (III-IV) and specific molecular subtypes. These findings demonstrate that the prognosis model based on tumor border characteristics provides reliable predictive value and correlates well with clinical characteristics, establishing it as a robust tool for assessing progression risk in breast cancer patients (Figure 5E).

### Favorable prognostic value of MBS across various subtypes

**FIGURE 6:**
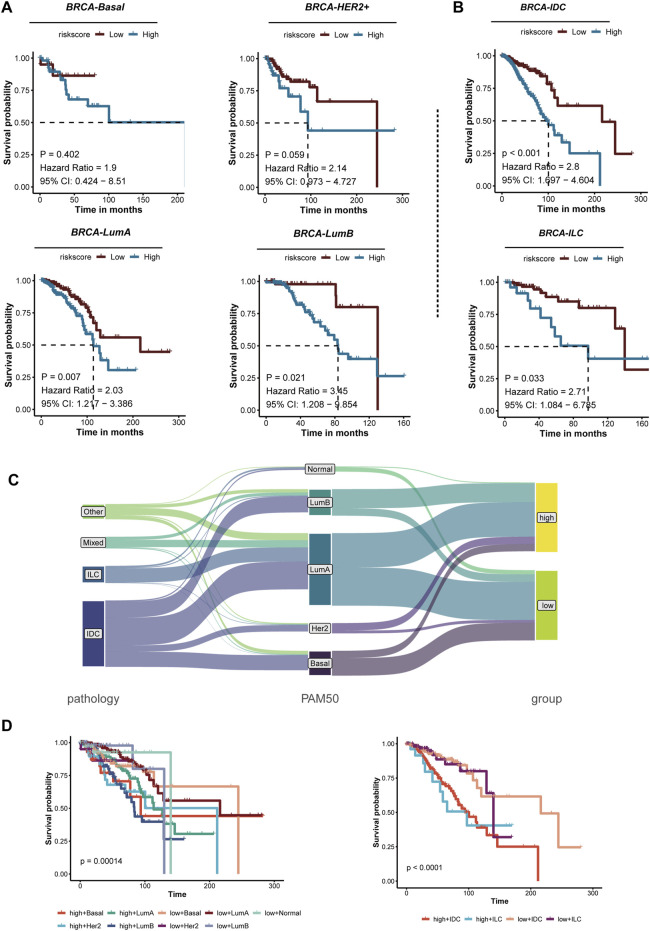
The prognosis of risk characteristics across various breast cancer subtypes is analyzed. **(A)** Kaplan-Meier survival analysis of different PAM50 molecular subtypes of breast cancer, including Basal, HER2^+^, Luminal A (LumA), and Luminal B (LumB), reveals that patients are categorized into high-risk and low-risk groups based on risk scores. Each panel presents the hazard ratio (HR), 95% confidence interval (CI), and *P*-values from the risk assessment. Notable differences in survival rates are observed between the LumA and LumB subtypes. **(B)** Kaplan-Meier survival analysis for breast cancer subtypes, specifically IDC and ILC, indicates that high-risk patients in both subtypes exhibit significantly poorer outcomes. **(C)** A schematic representation illustrates the relationship between breast cancer pathology, PAM50 molecular subtypes, and risk groups. The classification of patients into high-risk and low-risk groups based on different pathologies and molecular subtypes suggests a correlation with their risk characteristics. **(D)** Kaplan-Meier survival analysis compares high-risk and low-risk groups for both PAM50 subtypes (left) and pathological subtypes (right). The survival curves demonstrate that the risk characteristics effectively stratify patients into distinct subtypes, with high-risk patients consistently exhibiting poor prognoses.

Furthermore, we evaluated the prognostic across various subtypes of BRCA. Kaplan-Meier survival curves were generated for each major subtype, revealing significant differences in survival outcomes correlated with risk scores. BRCA-LumA (*P* = 0.007, HR = 2.03, 95% CI: 1.208–3.854), and BRCA-LumB (*P* = 0.021, HR = 3.5, 95% CI: 1.786–7.052). The results showed that the novel signature could refined Luminal BRCA: BRCA-LumA (*P* = 0.007, HR = 2.03, 95% CI: 1.208–3.854), and BRCA-LumB (*P* = 0.021, HR = 3.5, 95% CI: 1.786–7.052) (Figure 6A). Significant survival differences were noted in the BRCA-IDC subtype (*P* < 0.001, HR = 2.8, 95% CI: 1.697–4.604), Additionally, significant survival differences were observed in BRCA-ILC (*P* = 0.033, HR = 2.71, 95% CI: 1.084–6.745), with high-risk patients exhibiting significantly worse survival outcomes (Figure 6B). Most cases of IDC are categorized into the Her2, LumA, and LumB in PAM50 subtypes, while ILC cases are primarily classified as LumA and LumB. Notably, the high MBS subtype is predominantly composed of Luminal tumors. The low MBS subtypes mainly consist of LumA and Basal, with a lower proportion of LumB, which aligns with previous survival analyses. These findings suggest that the current grading system can still be further subdivided (Figure 6C). Additional survival analyses indicate that the High-MBS LumB subtype has the poorest prognosis, whereas the Low-MBS Basal subtype exhibits a more favorable outcome (*P* = 0.00014); in the context of pathological classification, prognosis is primarily associated with MBS(*P* < 0.0001) (Figure 6D). These results underscore the significance of MBS characteristics in breast cancer prognosis, highlighting the need for focused efforts in accurate prognosis and prediction.

### High-MBS score BRCA had poor response to adjuvant therapy

**FIGURE 7:**
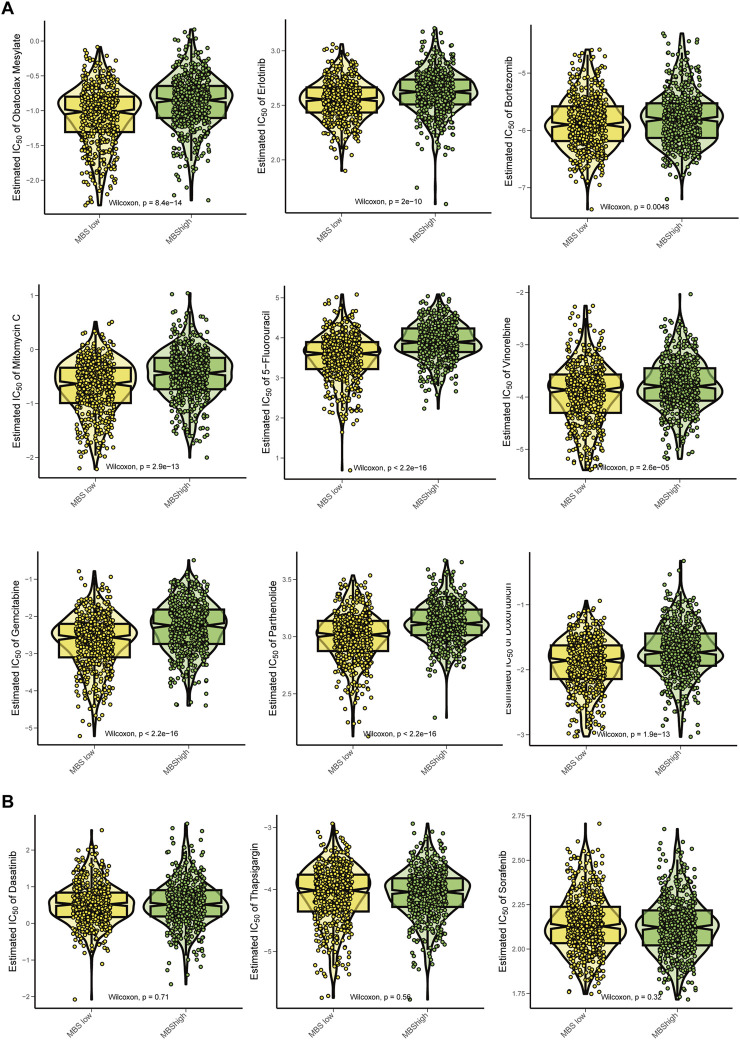
Chemotherapy sensitivity analysis for Malignant-boundary signature. **(A)** Comparison of the estimated effects of various chemotherapy drugs between the MBS-high group and the MBS-low group. The drugs analyzed include Oxaliplatin, Erlotinib, Bortezomib, Mitomycin C, 5-Fluorouracil, Vinorelbine, Gemcitabine and Pyrimethamine. We utilized the Wilcoxon test to assess statistical significance, displaying the P values in each panel. A lower IC50 value indicates higher drug sensitivity. **(B)** Comparison of IC50 of Dasatinib, Thapsigargin, and Sorafenib between MBS-high and MBS-low groups.

We calculated the IC50 values to compare the estimated drug sensitivities of the high-MBS and low-MBS groups to various compounds and performed Wilcoxon tests to assess the statistical significance of the differences between the two groups. Oxaliplatin, Erlotinib, and Bortezomib exhibited significantly lower IC50 values in the low-risk group, indicating that these patients are more sensitive to these drugs (P values: *P* = 8.4e^−14^, *P* = 2e^−16^, and *P* = 0.049). Mitomycin C, 5-Fluorouracil, and Vinorelbine also demonstrated significant differences, with low-risk patients showing greater sensitivity to these drugs (*P* = 2.9e^−13^, *P* = 2.2e^−16^, and *P* = 2.6e^−16^). This further underscores the disparity in drug sensitivity, as low-risk patients exhibit a trend of increased sensitivity to Gemcitabine and Pyrimethamine (*P* = 1.3e^−16^ and *P* = 9.9e^−13^) (Figure 7A). Additionally, Dasatinib, Thapsigargin, and Sorafenib demonstrated significant differences in drug sensitivity between high-risk and low-risk groups (Figure 7B). Overall, breast cancer characterized by high MBS traits displays reduced sensitivity to auxiliary treatments.

### Patients with high-MBS were unlikely to benefit from immunotherapy

**FIGURE 8:**
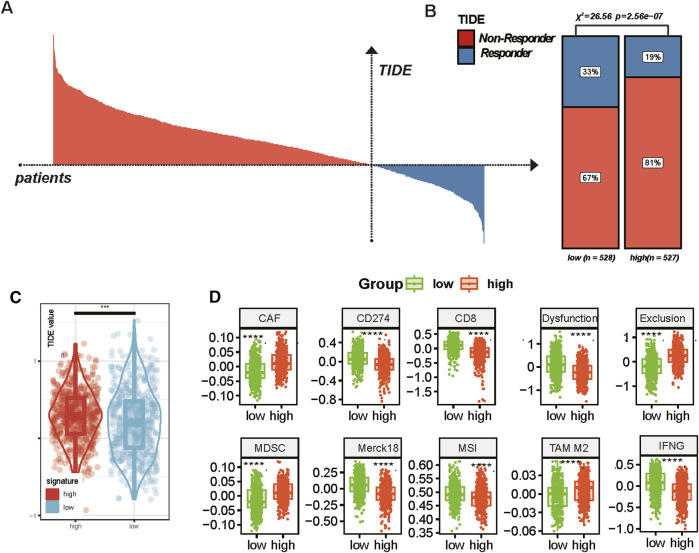
Tumor boundary characteristics and immune response in breast cancer. **(A)** Distribution of TIDE scores across all patients. Based on TIDE values, responders (blue) and non-responders (red) are clearly distinguished, indicating the predictive value of TIDE in assessing immune evasion in cancer treatment. **(B)** Proportion of responders and non-responders in low and high TIDE score groups. Chi-square analysis was used for statistical analysis. **(C)** Comparison of TIDE scores between the low and high MBS groups (* represents *P* < 0.05; ** represents *P* < 0.01; *** represents *P* < 0.001, **** represents *P* < 0.0001). **(D)** Comparison of expression profiles of various immune-related genes and pathways in the low and high signature groups. The box plots show the median and interquartile range (* represents *P* < 0.05; ** represents *P* < 0.01; *** represents *P* < 0.001, **** represents *P* < 0.0001).

The characteristics of the tumor microenvironment and treatment responses were analyzed using the TIDE model, which classifies patients based on their treatment response and evaluates responders and non-responders through TIDE scores. Furthermore, we examined the differences in responders between patients with high and low MBS (Figure 8A). The chi-square test revealed significant differences, with the high-MBS group comprising 81% non-responders and 19% responders, while the low-MBS tissue included 67% non-responders and 33% responders (chi-square value = 26.56, *P* = 2.56e^−07^) (Figure 8B). Compared to the low-characteristic group, the TIDE values in the high-characteristic group were significantly higher (*P* < 0.0001) (Figure 8C), indicating more severe immune dysfunction in the high-risk group. Additionally, we compared the expression profiles of key immune-related genes and pathways between the two groups, including CAF, CD274, CD8, dysfunction, exclusion, MDSC, Merck18, MSI, TAM M2, and IFNG. The box plots indicated significant differences in gene expression between the low-feature and high-feature groups, with the high-MBS group exhibiting markedly elevated CAF, exclusion, MDSC, and M2 scores (*P* < 0.0001) (Figure 8D), which is consistent with its phenotype representing a poorer immune response. Notably, the enrichment scores of CAF and M2 macrophages were significantly increased, aligning with previous analyses that suggest the barrier formed by CAF and M2 macrophages at the tumor boundary can promote tumor immune escape. In the low-MBS group, we observed a higher presence of immune activation markers, including MSI, IFNG, and CD8, which is consistent with previous research findings (*P* < 0.0001).

## Discussion

Tumor boundary research has long been a focal point in cancer studies, as these regions represent the interface between malignant and non-malignant tissues, often determining the progression and metastasis of cancer (Erickson et al., 2022). Traditionally, research has concentrated on the core of tumors, while the boundaries between tumors and surrounding tissues have received less attention. However, emerging studies underscore the complexity of these boundary areas, where the surrounding matrix and immune environment significantly influence prognosis (Qi et al., 2022; Liu et al., 2023). In breast cancer, the role of the boundaries between normal tissue and malignant growth remains unclear, especially regarding the intricate interactions among various cell types surrounding the tumor. This study aims to utilize spatial transcriptomic (ST) to create a molecular map of the tumor boundary, thereby providing a comprehensive analysis of gene expression in different tumor regions to address this knowledge gap.

In this study, we utilized various types of spatial transcription data from mammary cancer subtypes, including DCIS, IDC, ILC, and TNBC. We identified tumor boundaries that are enriched in genetic reprogramming, immune regulation, and cell migration associated with the ECM. These findings underscore the dynamic nature and heterogeneity of the tumor microenvironment. The tumor boundary serves as a site for active cellular interactions that promote cancer progression. Notably, genes associated with EMT, immune cell migration, and ECM remodeling are highly expressed at the tumor boundary, reinforcing the importance of this region in facilitating tumor invasion and metastasis. EMT facilitates tumor cells in breaching local tissue barriers by enabling them to acquire interstitial characteristics (Kalluri and Weinberg, 2009; Ariosa et al., 2021), such as enhanced migratory capabilities, increased invasiveness, and drug resistance (Hashemi et al., 2022). This process allows tumor cells to enter the bloodstream or lymphatic system, ultimately leading to the formation of metastases. EMT plays a crucial role in the metastasis of breast cancer (Chen et al., 2024). Consequently, understanding the mechanisms underlying EMT is of paramount importance for developing effective treatments for breast cancer, as both chemotherapy and targeted therapies are linked to the resistance exhibited by tumor cells. EMT can promote the characteristics of tumor cells that contribute to their resistance to chemotherapy drugs (Xu et al., 2017; Li et al., 2023). Furthermore, during the EMT process, the interaction between tumor cells and the ECM is intensified, complicating the ability of the immune system to identify and eliminate these cells, thereby further enhancing their capacity for escape (Zhou et al., 2019).

Our analysis also reveals a significant co-localization of CAFs and M2-like TAMs at the tumor boundary, indicating their synergistic role in supporting the microenvironment conducive to tumor growth. CAFs constitute a heterogeneous population that persists in the TME, significantly contributing to tumor progression. They are involved in tumor invasion, metastasis, immune evasion, and treatment resistance through various mechanisms. CAFs actively secrete ECM components, including collagen, fibronectin, and hyaluronic acid, which create a rigid tumor matrix that promotes cancer cell migration and invasion (Kalluri and Zeisberg, 2006). Furthermore, CAFs release growth factors such as TGF-β, VEGF, and FGF, which enhance EMT and tumor proliferation (Gascard and Tlsty, 2016). CAFs can also inhibit anti-tumor immunity by secreting cytokines such as IL-6 and TGF-β, leading to the recruitment of immunosuppressive cells like T regulatory cells and M2-like TAMs, thereby promoting immune evasion within the TME. Additionally, CAFs enhance drug resistance by forming a physical barrier that restricts drug penetration and secreting soluble factors that activate survival pathways in cancer cells. For instance, IL-6 secreted by CAFs can activate the JAK/STAT3 pathway, thereby reducing the sensitivity of breast cancer cells to chemotherapy. In estrogen receptor-positive breast cancer, CAFs communicate through paracrine signaling involving IL-6 and CXCL12 (Liu et al., 2019). M2-like TAMs secrete VEGF and PDGF, which enhance the formation of new blood vessels and support tumor growth and metastasis (Qian and Pollard, 2010). Additionally, M2 TAMs contribute to chemoresistance by secreting IL-10 and transforming TGF-β, which provide survival signals to cells and inhibit apoptosis induced by chemotherapeutic agents such as doxorubicin and paclitaxel. Furthermore, M2 TAMs promote resistance to endocrine therapy by actively activating the STAT3 and NF-κB pathways in ER^+^ breast cancer, thereby reducing sensitivity to hormone therapies (DeNardo et al., 2011). In conclusion, both CAF and M2-like TAM play critical roles in the progression of breast cancer and the development of drug resistance. The interaction between CAFs and M2 TAMs contributes to the remodeling of the ECM, promotes tumor angiogenesis, facilitates immune evasion, and enhances chemical resistance. Investigating the CAF-M2 interaction represents a promising strategy for improving breast cancer treatment. Future research should prioritize the development of combined therapies targeting both CAFs and TAM-like M2 TAMs to overcome resistance to chemotherapy, endocrine therapy, and immunotherapy.

In addition to the molecular insights provided by the spatial analysis, we have developed a prognostic model based on border-related genes. Through this model, we identified a set of genes capable of predicting patient survival outcomes with high precision. Notably, the MBS scores are closely associated with clinical outcomes; a high score indicates a poor prognosis. Importantly, the MBS score can predict survival across various breast cancer subtypes, including HER2-positive, luminal, and basal subtypes, underscoring the robustness of the model. Furthermore, the MBS score correlates with chemotherapy resistance, as patients with high MBS scores had limited opportunity to benefit from chemotherapy strategies. Some commonly used drugs, such as 5-FU and doxorubicin, may not benefit patients with high MBS. This suggests that tumor boundaries not only influence cancer progression but also play a crucial role in determining treatment responses. High-risk groups with elevated MBS scores represent a particularly challenging subset of breast cancer patients who may not respond well to conventional therapies, highlighting the urgent need for new treatment strategies targeting tumor boundaries.

However, this study does have several limitations that warrant consideration. Firstly, the spatial transcriptomic data is derived from a limited number of samples, which may not fully capture the heterogeneity of different breast cancer subtypes. While this study employs multiple datasets from various cohorts, further validation in larger and more diverse patient populations is necessary to confirm the stability of the identified biomarkers and prognostic models.

In conclusion, the tumor-stroma boundary plays a crucial role in tumor progression, metastasis, and immune evasion. The molecular characteristics of this region, revealed through spatial multi-omics analysis, can serve as diagnostic markers for risk stratification and the prediction of therapeutic response. Patients with high MBS scores have a poor prognosis and exhibit resistance to chemotherapy, indicating that such patients require intensified combination therapy rather than relying solely on standard chemotherapy. High MBS tumors demonstrate immune dysfunction and immune exclusion, which limit the efficacy of immune checkpoint inhibitors. Therefore, the MBS score aids in differentiating patients: those with low MBS may benefit from monotherapy with immune agents, while those with high MBS necessitate combined immune activation strategies to overcome resistance. Incorporating the MBS score into clinical decision-making can optimize treatment strategies based on the characteristics of the tumor microenvironment (TME). Targeting the interaction between CAF-M2 TAM at the tumor boundary represents a potential therapeutic direction. For instance, combining CAF-targeting drugs (such as TGF-β inhibitors and FAP inhibitors) with immune checkpoint inhibitors may reverse immune exclusion.

## Data Availability

Spatial transcriptome (ST) data of breast cancer was retrieved from GEO database (https://www.ncbi.nlm.nih.gov/geo/) and 10x Genomics official website (https://www.10xgenomics.com/): GSM6433610 from the GSE210616 dataset and GSM6177603 from the GSE203612 dataset. Data regarding gene expression and comprehensive clinical information for BRCA patients were sourced from The Cancer Genome Atlas (TCGA, https://portal.gdc.cancer.gov/).
