# Supplementary figures and images for: Spatial multi-omics analysis of tumor-stroma boundary cell features for predicting breast cancer progression and therapy response
Source: Front Cell Dev Biol. 2025 Mar 26;13:1570696. doi: 10.3389/fcell.2025.1570696 (PMC11979139; doi:10.3389/fcell.2025.1570696)

Patient 7# *DAPI* *CD163* *ACTA2* *FAP* *CD8*

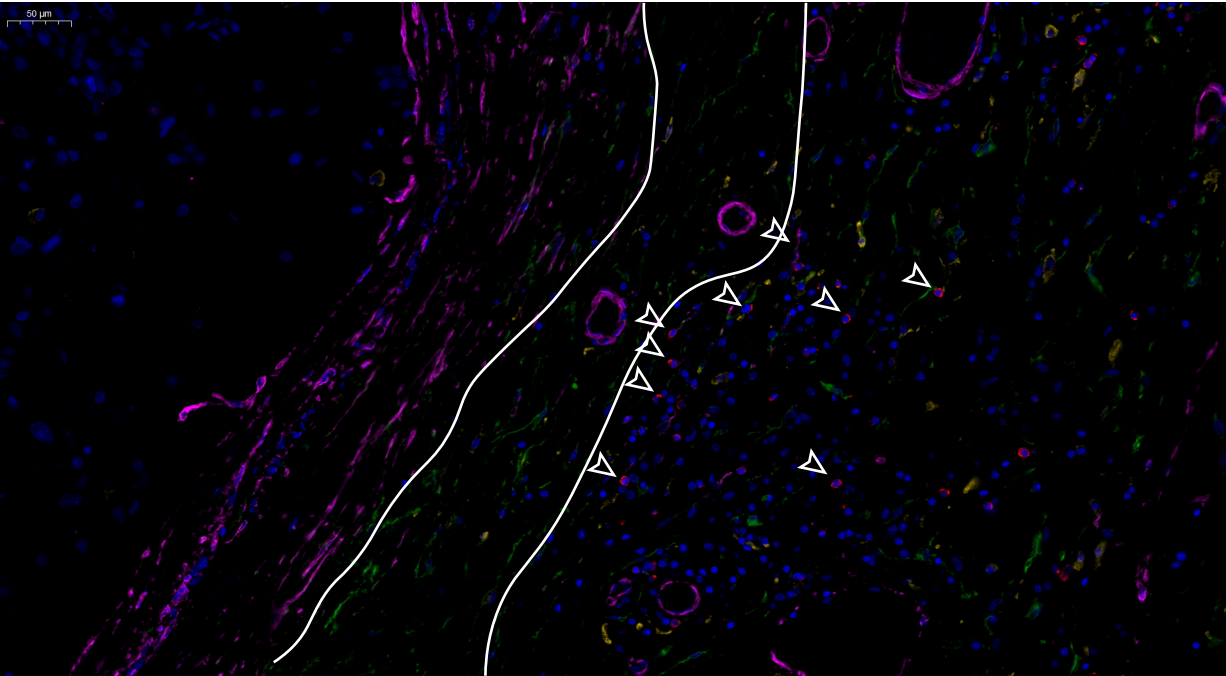

Patient 8# *DAPI* *CD163* *ACTA2* *FAP* *CD8*

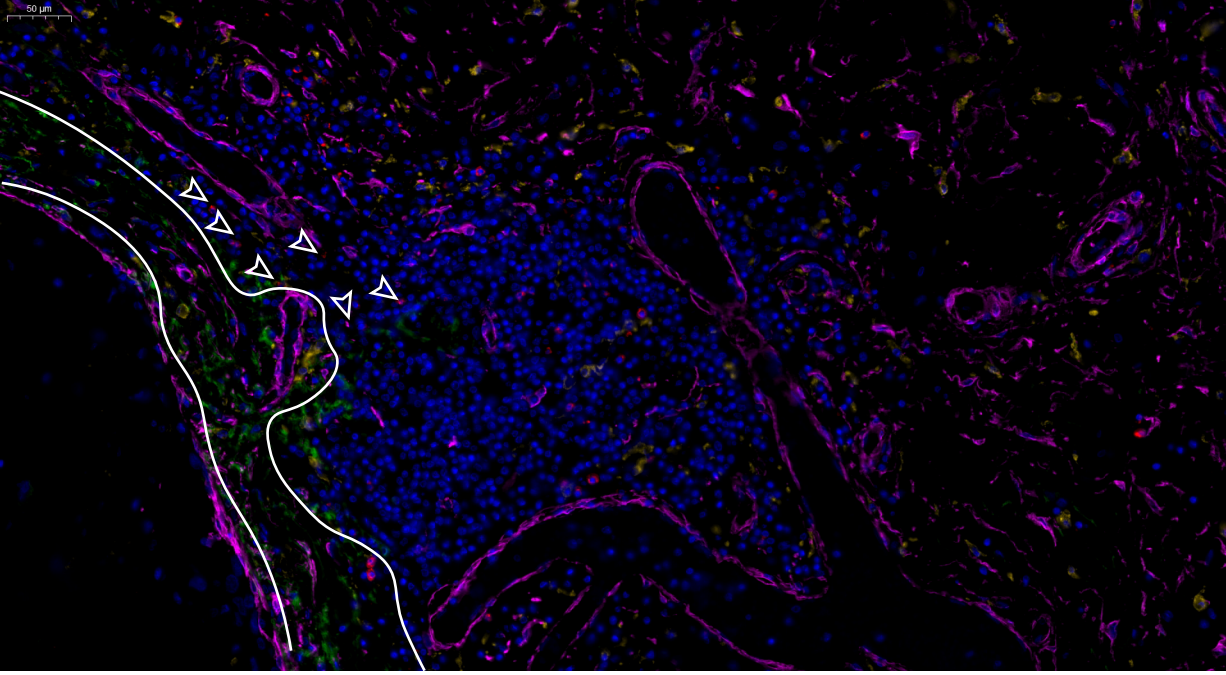

Supplementary Figure2

Supplement: Supplementary file 1 [file DataSheet2.pdf]
