# Supplementary material for: Spatial multi-omics analysis of tumor-stroma boundary cell features for predicting breast cancer progression and therapy response
Source: Front Cell Dev Biol. 2025 Mar 26;13:1570696. doi: 10.3389/fcell.2025.1570696 (PMC11979139; doi:10.3389/fcell.2025.1570696)
Supplement: Supplementary file 2 [file Table1.docx]

**Supplementary Table1: Upregulated genes in Bdy area across the four samples.**

|  | | | |
| --- | --- | --- | --- |
| **10x-BRCA** | **10x-BRCA2** | **GSM433610** | **GSM6177603** |
| ZYX | ZYX | ZYX | ZYX |
| ZNFX1 | ZFP36L2 | ZSWIM8 | ZNFX1 |
| ZMIZ1 | ZFP36L1 | ZNRD1 | ZMIZ1 |
| ZDHHC20 | YBX1 | ZNHIT1 | ZDHHC20 |
| YWHAH | WIPF1 | ZNF692 | YWHAH |
| XRCC5 | VWF | ZFP36 | XRCC5 |
| XAF1 | VIM | ZER1 | XAF1 |
| WIPF2 | VCAN | ZBED1 | WIPF2 |
| WIPF1 | TYROBP | YKT6 | WIPF1 |
| WDR1 | TYMP | YIF1A | WDR1 |
| WDFY1 | TXNIP | YBX1 | WDFY1 |
| WARS | TRBC2 | XAF1 | WARS |
| VWF | TRAC | WIPF1 | VWF |
| VIM | TPT1 | WDR6 | VIM |
| VDAC1 | TNC | WDR46 | VDAC1 |
| VASP | TMSB4X | WASHC1 | VASP |
| VASH1 | TIMP3 | VWF | VASH1 |
| VAMP5 | TIMP2 | VSIR | VAMP5 |
| USP18 | TIMP1 | VPS28 | USP18 |
| UNC5B | THY1 | VPS25 | UNC5B |
| UBD | THBS2 | VKORC1 | UBD |
| UBB | TGFBI | VIM | UBB |
| UACA | TAGLN | VCAN | UACA |
| TSTA3 | SULF1 | VASP | TSTA3 |
| TSPAN15 | SRGN | VASH1 | TSPAN15 |
| TRPS1 | SPI1 | VAMP5 | TRPS1 |
| TRIM29 | SPARCL1 | USF2 | TRIM29 |
| TRIM22 | SPARC | URM1 | TRIM22 |
| TPM2 | SNCG | UQCRC1 | TPM2 |
| TPM1 | SLC15A3 | UQCR11 | TPM1 |
| TPD52 | SH3BGRL3 | UQCC3 | TPD52 |
| TP53I11 | SFRP4 | UQCC2 | TP53I11 |
| TOP2A | SFRP2 | UPF1 | TOP2A |
| TNFSF10 | SERPING1 | UNC5B | TNFSF10 |
| TNFAIP3 | SERPINF1 | UNC45A | TNFAIP3 |
| TMEM258 | SERPINA3 | UNC13D | TMEM258 |
| TMEM204 | SERPINA1 | UBTF | TMEM204 |
| TMEM123 | SAT1 | UBL4A | TMEM123 |
| TIMP3 | SAMHD1 | UBE2M | TIMP3 |
| TIMP1 | S100A6 | UBC | TIMP1 |
| THY1 | S100A4 | UBB | THY1 |
| THRA | S100A10 | UBA7 | THRA |
| THBS1 | RPS9 | UBA52 | THBS1 |
| TGFBI | RPS3 | TYROBP | TGFBI |
| TGFB1I1 | RPS25 | TXNIP | TGFB1I1 |
| TGFB1 | RPL36 | TXNDC11 | TGFB1 |
| TFDP1 | RPL27A | TXN2 | TFDP1 |
| TFAP2B | RPL21 | TWF2 | TFAP2B |
| TCF4 | RPL18A | TUFM | TCF4 |
| TAP1 | RNASE1 | TUBB6 | TAP1 |
| TAGLN2 | RAC2 | TUBB4B | TAGLN2 |
| TAGLN | PTPRC | TUBB | TAGLN |
| TACSTD2 | PSAP | TUBA1C | TACSTD2 |
| TAB2 | PRSS23 | TUBA1B | TAB2 |
| SULF1 | PPP1R18 | TUBA1A | SULF1 |
| STOM | POSTN | TTYH3 | STOM |
| STARD3 | PLEKHO1 | TSTA3 | STARD3 |
| SRCIN1 | PCOLCE | TSSC4 | SRCIN1 |
| SPRY4 | NPC2 | TSPO | SPRY4 |
| SPRY1 | NNMT | TSPAN4 | SPRY1 |
| SPP1 | NAP1L1 | TSKU | SPP1 |
| SPINT2 | MYL9 | TRPC4AP | SPINT2 |
| SPI1 | MX1 | TRIR | SPI1 |
| SPATS2L | MT-ND5 | TRIP6 | SPATS2L |
| SPARCL1 | MT-ND4L | TRIM28 | SPARCL1 |
| SPARC | MT-ND4 | TRBC2 | SPARC |
| SOD1 | MT-ND3 | TRBC1 | SOD1 |
| SNCG | MT-ND2 | TRAPPC6A | SNCG |
| SMARCE1 | MT-ND1 | TRAPPC2L | SMARCE1 |
| SLPI | MT-CYB | TRAF7 | SLPI |
| SLC9A3R2 | MT-CO3 | TRAF4 | SLC9A3R2 |
| SKIL | MT-CO2 | TRAC | SKIL |
| SIPA1 | MT-CO1 | TRABD | SIPA1 |
| SHANK3 | MT-ATP6 | TPT1 | SHANK3 |
| SGK1 | MSN | TPSB2 | SGK1 |
| SET | MS4A6A | TPSAB1 | SET |
| SERPINB9 | MRC2 | TPM2 | SERPINB9 |
| SERINC2 | MMP9 | TPM1 | SERINC2 |
| SEPTIN11 | MMP2 | TPI1 | SEPTIN11 |
| SEMA3F | MGP | TP53I11 | SEMA3F |
| SELENOH | MFGE8 | TOMM40 | SELENOH |
| SDC1 | MARCKS | TOLLIP | SDC1 |
| SCGB2A2 | MALAT1 | TNS2 | SCGB2A2 |
| SCGB1D2 | LYZ | TNK2 | SCGB1D2 |
| SAMD9L | LUM | TNIP1 | SAMD9L |
| S100A2 | LTBP2 | TNFRSF1B | S100A2 |
| S100A16 | LTB | TNFRSF14 | S100A16 |
| S100A14 | LST1 | TNFRSF12A | S100A14 |
| S100A11 | LSP1 | TMSB4X | S100A11 |
| ROBO1 | LRP1 | TMEM8A | ROBO1 |
| RNF145 | LIMD2 | TMEM256 | RNF145 |
| RHOB | LGMN | TMEM250 | RHOB |
| RGS5 | LGALS9 | TMEM222 | RGS5 |
| RGS1 | LGALS3 | TMEM219 | RGS1 |
| RGCC | LGALS1 | TMEM208 | RGCC |
| RDH10 | LCP1 | TMEM205 | RDH10 |
| RARA | LAPTM5 | TMEM176B | RARA |
| RAMP3 | LAMB1 | TMEM176A | RAMP3 |
| RAB31 | KCTD12 | TMEM173 | RAB31 |
| RAB11A | JCHAIN | TMEM132A | RAB11A |
| QKI | ISLR | TMEM129 | QKI |
| PXDN | IL32 | TMEM119 | PXDN |
| PTPRG | IGLC3 | TMEM115 | PTPRG |
| PTPRF | IGLC2 | TMED9 | PTPRF |
| PTPRE | IGLC1 | TMED1 | PTPRE |
| PTPRB | IGKC | TMC6 | PTPRB |
| PTPA | IGHM | TMBIM1 | PTPA |
| PTP4A3 | IGHG4 | TIMP1 | PTP4A3 |
| PTMS | IGHG3 | TIMM8B | PTMS |
| PTK7 | IGHG2 | TIMM44 | PTK7 |
| PTK2 | IGHG1 | TIMM17B | PTK2 |
| PSME2 | IGHA1 | TIMM10 | PSME2 |
| PSMD3 | IGFBP7 | THY1 | PSMD3 |
| PSMB8 | IGFBP3 | THBS2 | PSMB8 |
| PSMB2 | IFI30 | THBS1 | PSMB2 |
| PSMB1 | IFI16 | THAP7 | PSMB1 |
| PSMA7 | ID3 | TGM2 | PSMA7 |
| PSMA5 | HSPG2 | TGFBR2 | PSMA5 |
| PSMA4 | HLA-E | TFE3 | PSMA4 |
| PRSS23 | HLA-DRB5 | TEX264 | PRSS23 |
| PROM2 | HLA-DRB1 | TCIRG1 | PROM2 |
| PREX1 | HLA-DRA | TCF25 | PREX1 |
| PPP2CB | HLA-DQB1 | TAZ | PPP2CB |
| PPM1F | HLA-DQA2 | TAX1BP3 | PPM1F |
| PPDPF | HLA-DQA1 | TAPBP | PPDPF |
| POSTN | HLA-DPB1 | TAP1 | POSTN |
| POLR2K | HLA-DPA1 | TAGLN2 | POLR2K |
| POLR2G | HLA-DMB | TAGLN | POLR2G |
| PODXL | HLA-DMA | SYTL1 | PODXL |
| PNP | HLA-C | SYMPK | PNP |
| PML | HLA-B | SUPT5H | PML |
| PMEPA1 | HLA-A | STX10 | PMEPA1 |
| PLXDC1 | HCLS1 | STUB1 | PLXDC1 |
| PLVAP | GSTP1 | STRN4 | PLVAP |
| PLSCR1 | GSN | STOML1 | PLSCR1 |
| PLPP1 | GPSM3 | STAT1 | PLPP1 |
| PLIN2 | GPNMB | STARD3 | PLIN2 |
| PLEKHO1 | GNAI2 | STAB1 | PLEKHO1 |
| PLEKHG2 | GMFG | ST14 | PLEKHG2 |
| PLAUR | GIMAP4 | SSSCA1 | PLAUR |
| PLAT | GAS6 | SSR4 | PLAT |
| PLAAT4 | FYB1 | SSNA1 | PLAAT4 |
| PITX1 | FXYD5 | SRRM2 | PITX1 |
| PILRB | FTL | SRM | PILRB |
| PIGR | FSTL1 | SRGN | PIGR |
| PICALM | FOS | SREBF1 | PICALM |
| PHLDB1 | FN1 | SRA1 | PHLDB1 |
| PHLDA1 | FLNA | SPTAN1 | PHLDA1 |
| PGAP3 | FGL2 | SPON2 | PGAP3 |
| PFN1 | FCGR3A | SPOCK2 | PFN1 |
| PEA15 | FCER1G | SPDEF | PEA15 |
| PDLIM7 | FBN1 | SPARCL1 | PDLIM7 |
| PDGFRB | FBLN1 | SPARC | PDGFRB |
| PDGFB | ENG | SNX17 | PDGFB |
| PDGFA | EMP3 | SNRPD2 | PDGFA |
| PDCD6 | EMILIN1 | SNRPB | PDCD6 |
| PCDH17 | EEF1G | SND1 | PCDH17 |
| PCDH12 | DPYSL2 | SNCG | PCDH12 |
| PALLD | DCN | SMTN | PALLD |
| ORMDL3 | CYBA | SMIM37 | ORMDL3 |
| OLR1 | CXCL9 | SMIM12 | OLR1 |
| OLFML2B | CXCL12 | SLIT3 | OLFML2B |
| OLFML2A | CTSS | SLC52A2 | OLFML2A |
| OAZ2 | CTSK | SLC3A2 | OAZ2 |
| OASL | CTSH | SLC39A13 | OASL |
| OAS3 | CTSD | SLC38A10 | OAS3 |
| OAS2 | CTSC | SLC35C2 | OAS2 |
| OAS1 | CTSB | SLC2A8 | OAS1 |
| NUDCD1 | CTHRC1 | SLC25A6 | NUDCD1 |
| NRP2 | CST1 | SLC25A39 | NRP2 |
| NREP | CSF1R | SLC25A29 | NREP |
| NRARP | COTL1 | SLC25A22 | NRARP |
| NPTN | CORO1A | SLC12A9 | NPTN |
| NOTCH4 | COMP | SLC12A7 | NOTCH4 |
| NOS3 | COL6A3 | SIVA1 | NOS3 |
| NID1 | COL6A2 | SIRT7 | NID1 |
| NES | COL6A1 | SIRT2 | NES |
| MYO1B | COL5A2 | SIGIRR | MYO1B |
| MYLK | COL5A1 | SHISA5 | MYLK |
| MYL9 | COL4A2 | SH3GLB2 | MYL9 |
| MYL12A | COL4A1 | SH3GL1 | MYL12A |
| MYH9 | COL3A1 | SH3BGRL3 | MYH9 |
| MYADM | COL1A2 | SFRP4 | MYADM |
| MX2 | COL1A1 | SFRP2 | MX2 |
| MX1 | COL18A1 | SF3B5 | MX1 |
| MUCL1 | COL15A1 | SERPINH1 | MUCL1 |
| MT2A | COL12A1 | SERPING1 | MT2A |
| MSN | CDH11 | SERPINF1 | MSN |
| MSL1 | CD74 | SERPINE2 | MSL1 |
| MRC2 | CD68 | SERPINE1 | MRC2 |
| MPRIP | CD52 | SERF2 | MPRIP |
| MMP14 | CD2 | SELENOO | MMP14 |
| MMP11 | CD163 | SELENON | MMP11 |
| MLPH | CD14 | SELENOH | MLPH |
| MLLT6 | CCNI | SEC61A1 | MLLT6 |
| MIEN1 | CCN2 | SDC4 | MIEN1 |
| MICAL2 | CCL5 | SCYL1 | MICAL2 |
| MFSD12 | CCDC80 | SCRN2 | MFSD12 |
| MFGE8 | CAVIN1 | SCAP | MFGE8 |
| MED24 | CAPG | SCAMP3 | MED24 |
| MDK | CALD1 | SCAMP2 | MDK |
| MCM7 | C9orf16 | SAA1 | MCM7 |
| MCF2L | C3 | S100P | MCF2L |
| MCAM | C1S | S100A6 | MCAM |
| MARK2 | C1R | S100A4 | MARK2 |
| MARCKSL1 | C1QC | S100A16 | MARCKSL1 |
| MARCKS | C1QB | S100A13 | MARCKS |
| MAP4 | C1QA | RPSA | MAP4 |
| MANF | BRI3 | RPS9 | MANF |
| MAL2 | BGN | RPS8 | MAL2 |
| LY6E | B2M | RPS5 | LY6E |
| LUM | ARL6IP5 | RPS29 | LUM |
| LTF | ARHGAP45 | RPS28 | LTF |
| LSP1 | APOE | RPS27A | LSP1 |
| LOXL2 | APOC1 | RPS27 | LOXL2 |
| LOXL1 | ANXA2 | RPS26 | LOXL1 |
| LMO7 | ANXA1 | RPS25 | LMO7 |
| LMO2 | AIF1 | RPS21 | LMO2 |
| LMNB2 | AEBP1 | RPS20 | LMNB2 |
| LLGL2 | ADA2 | RPS2 | LLGL2 |
| LGALS3BP | ACTA2 | RPS19 | LGALS3BP |
| LGALS1 | A2M | RPS18 | LGALS1 |
| LEF1 |  | RPS16 | LEF1 |
| LCP1 |  | RPS15A | LCP1 |
| LCN2 |  | RPS15 | LCN2 |
| LBP |  | RPS14 | LBP |
| LBH |  | RPS13 | LBH |
| LAPTM5 |  | RPS12 | LAPTM5 |
| LAPTM4B |  | RPS11 | LAPTM4B |
| LAP3 |  | RPS10 | LAP3 |
| LAMC2 |  | RPLP2 | LAMC2 |
| LAMC1 |  | RPLP1 | LAMC1 |
| LAMB3 |  | RPLP0 | LAMB3 |
| LAMB1 |  | RPL8 | LAMB1 |
| LAMA5 |  | RPL7A | LAMA5 |
| LAMA4 |  | RPL5 | LAMA4 |
| KTN1 |  | RPL4 | KTN1 |
| KRT8 |  | RPL38 | KRT8 |
| KRT7 |  | RPL36AL | KRT7 |
| KRT6B |  | RPL36 | KRT6B |
| KRT5 |  | RPL35 | KRT5 |
| KRT18 |  | RPL32 | KRT18 |
| KRT14 |  | RPL3 | KRT14 |
| KIAA1522 |  | RPL29 | KIAA1522 |
| KIAA1324 |  | RPL28 | KIAA1324 |
| KIAA1217 |  | RPL27A | KIAA1217 |
| KDR |  | RPL27 | KDR |
| JUP |  | RPL21 | JUP |
| JAG2 |  | RPL18A | JAG2 |
| JAG1 |  | RPL18 | JAG1 |
| ITGB6 |  | RPL15 | ITGB6 |
| ITGB1 |  | RPL13A | ITGB1 |
| ITGAV |  | RPL13 | ITGAV |
| ITGA5 |  | RPL12 | ITGA5 |
| ITGA3 |  | RPL11 | ITGA3 |
| ITGA11 |  | RPL10 | ITGA11 |
| ITGA1 |  | RNH1 | ITGA1 |
| ISG20 |  | RNF181 | ISG20 |
| ISG15 |  | RNASE1 | ISG15 |
| INSR |  | RHPN1 | INSR |
| INHBA |  | RHOT2 | INHBA |
| IGFBP7 |  | RHOG | IGFBP7 |
| IGFBP5 |  | RHOC | IGFBP5 |
| IGFBP3 |  | RGS5 | IGFBP3 |
| IFITM3 |  | RGS3 | IFITM3 |
| IFITM2 |  | RFXANK | IFITM2 |
| IFITM1 |  | RFNG | IFITM1 |
| IFIT3 |  | RELB | IFIT3 |
| IFIH1 |  | REEP4 | IFIH1 |
| IFI6 |  | RCN3 | IFI6 |
| IFI44L |  | RCE1 | IFI44L |
| IFI44 |  | RBM42 | IFI44 |
| IFI30 |  | RBM14 | IFI30 |
| IFI27 |  | RARRES3 | IFI27 |
| IFI16 |  | RARRES2 | IFI16 |
| IDO1 |  | RANGAP1 | IDO1 |
| IDH2 |  | RAMP1 | IDH2 |
| ID1 |  | RALGDS | ID1 |
| HSPG2 |  | RAC2 | HSPG2 |
| HSPE1 |  | RABL6 | HSPE1 |
| HOXB3 |  | RABAC1 | HOXB3 |
| HNRNPAB |  | RAB1B | HNRNPAB |
| HLA-F |  | R3HDM4 | HLA-F |
| HIST2H2AB | | QSOX1 | HIST2H2AB |
| HIST1H4H |  | QARS | HIST1H4H |
| HIST1H4E |  | PYCR1 | HIST1H4E |
| HIST1H4D |  | PYCARD | HIST1H4D |
| HIST1H4C |  | PTRHD1 | HIST1H4C |
| HIST1H4A |  | PTPN7 | HIST1H4A |
| HIST1H3H |  | PTPA | HIST1H3H |
| HIST1H3G |  | PTP4A3 | HIST1H3G |
| HIST1H2BO | | PTOV1 | HIST1H2BO |
| HIST1H2BN | | PTBP1 | HIST1H2BN |
| HIST1H2BH | | PSMG3 | HIST1H2BH |
| HIST1H2BG | | PSME2 | HIST1H2BG |
| HIST1H2BD | | PSMD8 | HIST1H2BD |
| HIST1H2BB | | PSMD2 | HIST1H2BB |
| HIST1H2AG | | PSMC3 | HIST1H2AG |
| HIST1H2AD | | PSMB9 | HIST1H2AD |
| HIST1H1E |  | PSMB8 | HIST1H1E |
| HIST1H1B |  | PSMB6 | HIST1H1B |
| HES4 |  | PSMB5 | HES4 |
| HERC6 |  | PSMB3 | HERC6 |
| HERC5 |  | PSMB10 | HERC5 |
| HECW2 |  | PRSS8 | HECW2 |
| H1F0 |  | PRRC2A | H1F0 |
| GTPBP4 |  | PRPF31 | GTPBP4 |
| GSTO1 |  | PRMT1 | GSTO1 |
| GSN |  | PRKACA | GSN |
| GSDMB |  | PRG4 | GSDMB |
| GREM1 |  | PRELID1 | GREM1 |
| GNLY |  | PRDX5 | GNLY |
| GNAS |  | PRAF2 | GNAS |
| GLIPR2 |  | PQLC1 | GLIPR2 |
| GJC1 |  | PPP6R1 | GJC1 |
| GJA1 |  | PPP4C | GJA1 |
| GCHFR |  | PPP2R1A | GCHFR |
| GBP5 |  | PPP1R18 | GBP5 |
| GBP4 |  | PPP1R14B | GBP4 |
| GBP2 |  | PPP1R12C | GBP2 |
| GBP1 |  | PPIB | GBP1 |
| GATA3 |  | POSTN | GATA3 |
| GASK1B |  | POR | GASK1B |
| GAS6 |  | POLR2F | GAS6 |
| GALNT6 |  | POLR2E | GALNT6 |
| GABRE |  | POLL | GABRE |
| FSTL1 |  | PML | FSTL1 |
| FPR3 |  | PLXND1 | FPR3 |
| FOXA1 |  | PLXNB2 | FOXA1 |
| FNDC3B |  | PLXNB1 | FNDC3B |
| FN1 |  | PLVAP | FN1 |
| FMNL3 |  | PLTP | FMNL3 |
| FLNA |  | PLOD1 | FLNA |
| FKBP3 |  | PLIN3 | FKBP3 |
| FILIP1L |  | PLEKHO1 | FILIP1L |
| FHL3 |  | PLEKHM2 | FHL3 |
| FGL2 |  | PLEKHJ1 | FGL2 |
| FGG |  | PLEC | FGG |
| FGB |  | PLD3 | FGB |
| FCGR3A |  | PLAUR | FCGR3A |
| FCER1G |  | PLA2G15 | FCER1G |
| FBN1 |  | PKM | FBN1 |
| FADS2 |  | PINK1 | FADS2 |
| F3 |  | PIH1D1 | F3 |
| F2R |  | PIGQ | F2R |
| ETS1 |  | PIEZO1 | ETS1 |
| ESRP1 |  | PHRF1 | ESRP1 |
| ESAM |  | PHPT1 | ESAM |
| ERH |  | PHLDB1 | ERH |
| ERBB2 |  | PHLDA3 | ERBB2 |
| EPSTI1 |  | PHF1 | EPSTI1 |
| EPCAM |  | PHB | EPCAM |
| ENTPD1 |  | PGLS | ENTPD1 |
| ENG |  | PGAP3 | ENG |
| ENC1 |  | PFN1 | ENC1 |
| EHD4 |  | PFKL | EHD4 |
| EFHD2 |  | PFDN5 | EFHD2 |
| EFHD1 |  | PEMT | EFHD1 |
| EDNRA |  | PECAM1 | EDNRA |
| ECE1 |  | PDXK | ECE1 |
| DYNC1I2 |  | PDRG1 | DYNC1I2 |
| DUSP6 |  | PDLIM2 | DUSP6 |
| DSTN |  | PDGFRB | DSTN |
| DPYSL3 |  | PCOLCE | DPYSL3 |
| DLC1 |  | PAXX | DLC1 |
| DKK3 |  | PALLD | DKK3 |
| DHCR24 |  | PAFAH1B3 | DHCR24 |
| DEK |  | P4HB | DEK |
| DDX60L |  | P3H3 | DDX60L |
| DDX58 |  | P3H1 | DDX58 |
| DDR1 |  | OXLD1 | DDR1 |
| DDB1 |  | OTUB1 | DDB1 |
| DBI |  | OST4 | DBI |
| CXCL9 |  | OS9 | CXCL9 |
| CXCL14 |  | OLFML3 | CXCL14 |
| CXCL11 |  | OGDH | CXCL11 |
| CXCL10 |  | OBP2B | CXCL10 |
| CTSB |  | OAZ1 | CTSB |
| CTNND1 |  | OAF | CTNND1 |
| CTNNB1 |  | NUMA1 | CTNNB1 |
| CST3 |  | NUDT22 | CST3 |
| CST1 |  | NUDT16L1 | CST1 |
| CRYBG1 |  | NUDT1 | CRYBG1 |
| CRISP3 |  | NUDCD3 | CRISP3 |
| CRIP2 |  | NUCB1 | CRIP2 |
| CRIP1 |  | NUBP2 | CRIP1 |
| CRABP2 |  | NT5C | CRABP2 |
| CPD |  | NSUN5 | CPD |
| CP |  | NRBP1 | CP |
| COX6C |  | NR4A1 | COX6C |
| COTL1 |  | NPDC1 | COTL1 |
| CORO1C |  | NPC2 | CORO1C |
| COL8A2 |  | NOXA1 | COL8A2 |
| COL6A2 |  | NOSIP | COL6A2 |
| COL5A2 |  | NOC2L | COL5A2 |
| COL5A1 |  | NNMT | COL5A1 |
| COL4A2 |  | NINJ1 | COL4A2 |
| COL4A1 |  | NES | COL4A1 |
| COL1A2 |  | NECAP2 | COL1A2 |
| COL1A1 |  | NDUFV1 | COL1A1 |
| COL18A1 |  | NDUFS8 | COL18A1 |
| COL15A1 |  | NDUFS7 | COL15A1 |
| COL12A1 |  | NDUFS6 | COL12A1 |
| COL11A1 |  | NDUFB7 | COL11A1 |
| COL10A1 |  | NDUFB2 | COL10A1 |
| CNN2 |  | NDUFB11 | CNN2 |
| CMPK2 |  | NDUFAF3 | CMPK2 |
| CLIP1 |  | NDUFA4L2 | CLIP1 |
| CLIC4 |  | NDUFA13 | CLIC4 |
| CLIC1 |  | NDUFA11 | CLIC1 |
| CLDN4 |  | NCLN | CLDN4 |
| CHN1 |  | NBL1 | CHN1 |
| CFB |  | NARFL | CFB |
| CDH5 |  | NAPRT | CDH5 |
| CDH11 |  | NAGLU | CDH11 |
| CDC6 |  | NAGK | CDC6 |
| CD93 |  | NAB2 | CD93 |
| CD74 |  | NAA60 | CD74 |
| CD34 |  | NAA10 | CD34 |
| CD24 |  | MYO9B | CD24 |
| CD200 |  | MYO1F | CD200 |
| CCT7 |  | MYO1C | CCT7 |
| CCND2 |  | MYL9 | CCND2 |
| CCN2 |  | MYL6B | CCN2 |
| CCL5 |  | MYL6 | CCL5 |
| CCL2 |  | MYH9 | CCL2 |
| CAVIN3 |  | MYDGF | CAVIN3 |
| CAVIN1 |  | MXRA8 | CAVIN1 |
| CAV2 |  | MXD3 | CAV2 |
| CASC3 |  | MVP | CASC3 |
| CAPN13 |  | MVB12A | CAPN13 |
| CAP1 |  | MUC1 | CAP1 |
| CANT1 |  | MTX1 | CANT1 |
| CALD1 |  | MTG2 | CALD1 |
| CALCRL |  | MT2A | CALCRL |
| C15orf48 |  | MSRB1 | C15orf48 |
| BST2 |  | MSN | BST2 |
| BMP8A |  | MS4A6A | BMP8A |
| BMP1 |  | MRPS12 | BMP1 |
| BLMH |  | MRPL55 | BLMH |
| BGN |  | MRPL54 | BGN |
| BEX3 |  | MRPL4 | BEX3 |
| AZGP1 |  | MRPL12 | AZGP1 |
| ATP5PD |  | MRC2 | ATP5PD |
| ATP5MF |  | MMP9 | ATP5MF |
| ATP5F1B |  | MMP3 | ATP5F1B |
| ATP1B1 |  | MMP2 | ATP1B1 |
| ATG5 |  | MINK1 | ATG5 |
| ATF5 |  | MICALL2 | ATF5 |
| ASS1 |  | MICAL1 | ASS1 |
| ASPH |  | MGMT | ASPH |
| ARPC2 |  | MGAT1 | ARPC2 |
| ARPC1B |  | MFSD10 | ARPC1B |
| ARPC1A |  | MFGE8 | ARPC1A |
| ARHGDIB |  | MFAP5 | ARHGDIB |
| ARHGAP31 | | MFAP4 | ARHGAP31 |
| ARF3 |  | MFAP2 | ARF3 |
| APP |  | MED15 | APP |
| APLNR |  | MDK | APLNR |
| APBB2 |  | MCM7 | APBB2 |
| ANXA5 |  | MCAM | ANXA5 |
| ANTXR1 |  | MBOAT7 | ANTXR1 |
| ANPEP |  | MBD3 | ANPEP |
| ANO1 |  | MAZ | ANO1 |
| AGRN |  | MARCKS | AGRN |
| AFAP1L2 |  | MAPKAPK3 | AFAP1L2 |
| AFAP1L1 |  | MAP4 | AFAP1L1 |
| AEBP1 |  | MAP2K2 | AEBP1 |
| ADGRF5 |  | MAN2C1 | ADGRF5 |
| ADAMTSL2 | | MAN1B1 | ADAMTSL2 |
| ADAMTS4 |  | MAF1 | ADAMTS4 |
| ADAMTS2 |  | MAF | ADAMTS2 |
| ADAMTS12 | | MADD | ADAMTS12 |
| ACTR3 |  | LZTS2 | ACTR3 |
| ACTN1 |  | LYZ | ACTN1 |
| ACTG2 |  | LYPLA2 | ACTG2 |
| ACTB |  | LY6E | ACTB |
| ACTA2 |  | LUM | ACTA2 |
| ABRACL |  | LTBP2 | ABRACL |
| A2M |  | LTBP1 | A2M |
|  |  | LST1 | GABRE |
|  |  | LSP1 |  |
|  |  | LSM7 |  |
|  |  | LRRC32 |  |
|  |  | LRPAP1 |  |
|  |  | LRP1 |  |
|  |  | LMNA |  |
|  |  | LMF2 |  |
|  |  | LMAN2 |  |
|  |  | LIMD2 |  |
|  |  | LGALS3BP |  |
|  |  | LGALS1 |  |
|  |  | LDHB |  |
|  |  | LCP1 |  |
|  |  | LBH |  |
|  |  | LAPTM5 |  |
|  |  | LAMTOR4 |  |
|  |  | LAMTOR1 |  |
|  |  | LAMB2 |  |
|  |  | LAMB1 |  |
|  |  | LAMA4 |  |
|  |  | KRT8 |  |
|  |  | KRT7 |  |
|  |  | KRT19 |  |
|  |  | KRT18 |  |
|  |  | KIFC3 |  |
|  |  | KIF12 |  |
|  |  | KCTD12 |  |
|  |  | JUP |  |
|  |  | JUNB |  |
|  |  | JCHAIN |  |
|  |  | JAG1 |  |
|  |  | ITGB4 |  |
|  |  | ITGB2 |  |
|  |  | ITGA7 |  |
|  |  | ITGA5 |  |
|  |  | ISYNA1 |  |
|  |  | ISOC2 |  |
|  |  | ISLR |  |
|  |  | ISG15 |  |
|  |  | IRF3 |  |
|  |  | IRF1 |  |
|  |  | INTS11 |  |
|  |  | INF2 |  |
|  |  | IL32 |  |
|  |  | IGSF8 |  |
|  |  | IGLC3 |  |
|  |  | IGLC2 |  |
|  |  | IGKC |  |
|  |  | IGHGP |  |
|  |  | IGHG3 |  |
|  |  | IGHG2 |  |
|  |  | IGHG1 |  |
|  |  | IGFBP7 |  |
|  |  | IGFBP4 |  |
|  |  | IGFBP3 |  |
|  |  | IGFBP2 |  |
|  |  | IFITM3 |  |
|  |  | IFITM2 |  |
|  |  | IFI35 |  |
|  |  | IFI27L2 |  |
|  |  | IFI27 |  |
|  |  | IFI16 |  |
|  |  | IER3 |  |
|  |  | IER2 |  |
|  |  | IDH3G |  |
|  |  | IDH2 |  |
|  |  | ID3 |  |
|  |  | ICAM2 |  |
|  |  | HYAL2 |  |
|  |  | HTRA3 |  |
|  |  | HSPG2 |  |
|  |  | HSPB1 |  |
|  |  | HSD17B10 | |
|  |  | HM13 |  |
|  |  | HLA-F |  |
|  |  | HLA-E |  |
|  |  | HLA-DRB5 | |
|  |  | HLA-DRB1 | |
|  |  | HLA-DRA |  |
|  |  | HLA-DQB1 | |
|  |  | HLA-DQA1 | |
|  |  | HLA-DPB1 | |
|  |  | HLA-DPA1 | |
|  |  | HLA-DMB |  |
|  |  | HLA-DMA | |
|  |  | HLA-C |  |
|  |  | HLA-B |  |
|  |  | HLA-A |  |
|  |  | HIGD2A |  |
|  |  | HGS |  |
|  |  | HDGF |  |
|  |  | HDAC7 |  |
|  |  | HDAC11 |  |
|  |  | HCST |  |
|  |  | HCLS1 |  |
|  |  | HADHA |  |
|  |  | H2AFJ |  |
|  |  | GYPC |  |
|  |  | GUK1 |  |
|  |  | GSTP1 |  |
|  |  | GSTO1 |  |
|  |  | GSTK1 |  |
|  |  | GSN |  |
|  |  | GRN |  |
|  |  | GRB7 |  |
|  |  | GRAMD1A | |
|  |  | GPX4 |  |
|  |  | GPS1 |  |
|  |  | GPR108 |  |
|  |  | GPI |  |
|  |  | GPD1 |  |
|  |  | GPC1 |  |
|  |  | GPAA1 |  |
|  |  | GNG11 |  |
|  |  | GNB2 |  |
|  |  | GNAI2 |  |
|  |  | GMPPA |  |
|  |  | GMFG |  |
|  |  | GLIS2 |  |
|  |  | GLIPR2 |  |
|  |  | GLG1 |  |
|  |  | GJA4 |  |
|  |  | GIPC1 |  |
|  |  | GIMAP7 |  |
|  |  | GBA |  |
|  |  | GAS6 |  |
|  |  | GAPDH |  |
|  |  | GADD45GIP1 | |
|  |  | GADD45B |  |
|  |  | GAA |  |
|  |  | G6PC3 |  |
|  |  | G0S2 |  |
|  |  | FZR1 |  |
|  |  | FYB1 |  |
|  |  | FXYD5 |  |
|  |  | FXR2 |  |
|  |  | FUOM |  |
|  |  | FTL |  |
|  |  | FTH1 |  |
|  |  | FSTL1 |  |
|  |  | FSCN1 |  |
|  |  | FOS |  |
|  |  | FN1 |  |
|  |  | FMNL1 |  |
|  |  | FLII |  |
|  |  | FKBP11 |  |
|  |  | FKBP10 |  |
|  |  | FIS1 |  |
|  |  | FIBP |  |
|  |  | FHL3 |  |
|  |  | FCGRT |  |
|  |  | FCER1G |  |
|  |  | FBXW5 |  |
|  |  | FBRS |  |
|  |  | FBN1 |  |
|  |  | FBLN5 |  |
|  |  | FBLN2 |  |
|  |  | FBLN1 |  |
|  |  | FAU |  |
|  |  | FASTK |  |
|  |  | FARSA |  |
|  |  | FAP |  |
|  |  | FAM96B |  |
|  |  | FAM50A |  |
|  |  | FAM32A |  |
|  |  | FAM20C |  |
|  |  | FAM193B |  |
|  |  | FAM129B |  |
|  |  | FADS3 |  |
|  |  | FABP5 |  |
|  |  | FABP4 |  |
|  |  | F13A1 |  |
|  |  | ETHE1 |  |
|  |  | ETFB |  |
|  |  | ESAM |  |
|  |  | ERF |  |
|  |  | ERCC1 |  |
|  |  | ERBB2 |  |
|  |  | EPS8L2 |  |
|  |  | EPN1 |  |
|  |  | EPHA1 |  |
|  |  | ENTPD6 |  |
|  |  | ENO1 |  |
|  |  | ENGASE |  |
|  |  | ENG |  |
|  |  | EMP3 |  |
|  |  | ELOVL1 |  |
|  |  | ELOB |  |
|  |  | ELN |  |
|  |  | ELF3 |  |
|  |  | EIF6 |  |
|  |  | EIF5A |  |
|  |  | EIF4G1 |  |
|  |  | EIF3G |  |
|  |  | EHD1 |  |
|  |  | EGR1 |  |
|  |  | EGFL7 |  |
|  |  | EFEMP2 |  |
|  |  | EEF2 |  |
|  |  | EEF1D |  |
|  |  | EDF1 |  |
|  |  | ECSCR |  |
|  |  | ECH1 |  |
|  |  | ECE1 |  |
|  |  | DTX2 |  |
|  |  | DRAP1 |  |
|  |  | DPT |  |
|  |  | DPP9 |  |
|  |  | DPP7 |  |
|  |  | DPP3 |  |
|  |  | DNM2 |  |
|  |  | DNAJB2 |  |
|  |  | DMAC1 |  |
|  |  | DHRS4L2 |  |
|  |  | DHRS3 |  |
|  |  | DGKZ |  |
|  |  | DDX56 |  |
|  |  | DDX39A |  |
|  |  | DDT |  |
|  |  | DDR1 |  |
|  |  | DDIT4 |  |
|  |  | DDAH2 |  |
|  |  | DCXR |  |
|  |  | DCTPP1 |  |
|  |  | DCN |  |
|  |  | DBNL |  |
|  |  | DAP |  |
|  |  | CYTOR |  |
|  |  | CYR61 |  |
|  |  | CYP27A1 |  |
|  |  | CYHR1 |  |
|  |  | CYC1 |  |
|  |  | CYBC1 |  |
|  |  | CYB5R3 |  |
|  |  | CYB561D2 |  |
|  |  | CXCL9 |  |
|  |  | CXCL12 |  |
|  |  | CUTA |  |
|  |  | CUL7 |  |
|  |  | CTSK |  |
|  |  | CTSD |  |
|  |  | CTSA |  |
|  |  | CTHRC1 |  |
|  |  | CTGF |  |
|  |  | CTDSP1 |  |
|  |  | CST7 |  |
|  |  | CST3 |  |
|  |  | CSRP1 |  |
|  |  | CSRNP1 |  |
|  |  | CSK |  |
|  |  | CSF1R |  |
|  |  | CRISPLD2 |  |
|  |  | CRELD1 |  |
|  |  | COX8A |  |
|  |  | COX7A1 |  |
|  |  | COX5B |  |
|  |  | COX4I1 |  |
|  |  | COTL1 |  |
|  |  | CORO1B |  |
|  |  | CORO1A |  |
|  |  | COPS9 |  |
|  |  | COPE |  |
|  |  | COMP |  |
|  |  | COL8A1 |  |
|  |  | COL6A3 |  |
|  |  | COL6A2 |  |
|  |  | COL6A1 |  |
|  |  | COL5A2 |  |
|  |  | COL5A1 |  |
|  |  | COL4A2 |  |
|  |  | COL4A1 |  |
|  |  | COL3A1 |  |
|  |  | COL1A2 |  |
|  |  | COL1A1 |  |
|  |  | COL18A1 |  |
|  |  | COL16A1 |  |
|  |  | COL15A1 |  |
|  |  | COL14A1 |  |
|  |  | COL12A1 |  |
|  |  | CNPY3 |  |
|  |  | CNN2 |  |
|  |  | CMTM3 |  |
|  |  | CLUH |  |
|  |  | CLTB |  |
|  |  | CLPTM1 |  |
|  |  | CLK3 |  |
|  |  | CLIC1 |  |
|  |  | CLDN4 |  |
|  |  | CLCN7 |  |
|  |  | CIRBP |  |
|  |  | CILP |  |
|  |  | CIB1 |  |
|  |  | CHRD |  |
|  |  | CHPF2 |  |
|  |  | CHPF |  |
|  |  | CHID1 |  |
|  |  | CHD3 |  |
|  |  | CHCHD5 |  |
|  |  | CERCAM |  |
|  |  | CDK10 |  |
|  |  | CDH5 |  |
|  |  | CDH11 |  |
|  |  | CDC37 |  |
|  |  | CD99 |  |
|  |  | CD81 |  |
|  |  | CD74 |  |
|  |  | CD68 |  |
|  |  | CD52 |  |
|  |  | CD4 |  |
|  |  | CD3E |  |
|  |  | CD36 |  |
|  |  | CD34 |  |
|  |  | CD2BP2 |  |
|  |  | CD2 |  |
|  |  | CD151 |  |
|  |  | CD14 |  |
|  |  | CCS |  |
|  |  | CCM2 |  |
|  |  | CCL5 |  |
|  |  | CCL2 |  |
|  |  | CCDC80 |  |
|  |  | CCDC57 |  |
|  |  | CCDC137 |  |
|  |  | CCDC12 |  |
|  |  | CCAR2 |  |
|  |  | CAVIN1 |  |
|  |  | CAV1 |  |
|  |  | CASKIN2 |  |
|  |  | CARHSP1 |  |
|  |  | CARD19 |  |
|  |  | CAPZB |  |
|  |  | CAPS |  |
|  |  | CAPN1 |  |
|  |  | CAPG |  |
|  |  | CALR |  |
|  |  | CALHM2 |  |
|  |  | CALD1 |  |
|  |  | CABIN1 |  |
|  |  | C9orf16 |  |
|  |  | C7orf50 |  |
|  |  | C3 |  |
|  |  | C1S |  |
|  |  | C1R |  |
|  |  | C1QC |  |
|  |  | C1QB |  |
|  |  | C1QA |  |
|  |  | C1orf162 |  |
|  |  | C19orf70 |  |
|  |  | C19orf66 |  |
|  |  | C19orf53 |  |
|  |  | C19orf25 |  |
|  |  | C15orf39 |  |
|  |  | C12orf57 |  |
|  |  | BST2 |  |
|  |  | BRMS1 |  |
|  |  | BRI3 |  |
|  |  | BRAT1 |  |
|  |  | BOK |  |
|  |  | BLVRB |  |
|  |  | BLOC1S1 |  |
|  |  | BGN |  |
|  |  | BCR |  |
|  |  | BCL3 |  |
|  |  | BCAR1 |  |
|  |  | BAX |  |
|  |  | BAG6 |  |
|  |  | BAD |  |
|  |  | B4GALT7 |  |
|  |  | B2M |  |
|  |  | AXL |  |
|  |  | AURKAIP1 |  |
|  |  | AUP1 |  |
|  |  | ATP6V1F |  |
|  |  | ATP6V0E2 |  |
|  |  | ATP6V0B |  |
|  |  | ATP5MC1 |  |
|  |  | ATP13A1 |  |
|  |  | ATOX1 |  |
|  |  | ARRDC2 |  |
|  |  | ARRDC1 |  |
|  |  | ARL6IP4 |  |
|  |  | ARL2 |  |
|  |  | ARID5A |  |
|  |  | ARHGEF1 |  |
|  |  | ARHGDIB |  |
|  |  | ARHGDIA |  |
|  |  | ARHGAP45 | |
|  |  | ARHGAP4 |  |
|  |  | ARHGAP1 |  |
|  |  | ARFGAP1 |  |
|  |  | ARF5 |  |
|  |  | ARAP1 |  |
|  |  | APRT |  |
|  |  | AP2S1 |  |
|  |  | AP2M1 |  |
|  |  | AP1M1 |  |
|  |  | AP1B1 |  |
|  |  | ANXA1 |  |
|  |  | ANO9 |  |
|  |  | ANGPTL2 |  |
|  |  | ANAPC11 |  |
|  |  | AKT1S1 |  |
|  |  | AKR1B1 |  |
|  |  | AKR1A1 |  |
|  |  | AIF1 |  |
|  |  | AGTRAP |  |
|  |  | AGRN |  |
|  |  | AGPAT1 |  |
|  |  | AGAP3 |  |
|  |  | AES |  |
|  |  | AEBP1 |  |
|  |  | ADRM1 |  |
|  |  | ADIRF |  |
|  |  | ADGRF5 |  |
|  |  | ADAM15 |  |
|  |  | ACTR1A |  |
|  |  | ACTN4 |  |
|  |  | ACTB |  |
|  |  | ACTA2 |  |
|  |  | ACAP1 |  |
|  |  | ACADVL |  |
|  |  | ABTB1 |  |
|  |  | ABR |  |
|  |  | ABCA3 |  |
|  |  | ABCA2 |  |
|  |  | A2M |  |
