# Supplementary material for: Spatial multi-omics analysis of tumor-stroma boundary cell features for predicting breast cancer progression and therapy response
Source: Front Cell Dev Biol. 2025 Mar 26;13:1570696. doi: 10.3389/fcell.2025.1570696 (PMC11979139; doi:10.3389/fcell.2025.1570696)
Supplement: Supplementary file 3 [file Table2.docx]

|  | **Genes** |
| --- | --- |
| **ECM*** | ZYX, WIPF1, VWF, VIM, TIMP1, THY1, TAGLN, SPARCL1, SPARC, SNCG, POSTN, PLEKHO1, MYL9, MSN, MRC2, MFGE8, MARCKS, LUM, LSP1, LGALS1, LCP1, LAPTM5, LAMB1, IGFBP7, IGFBP3, IFI16, HSPG2, GSN, GAS6, FSTL1, FN1, FCER1G, FBN1, ENG, CXCL9, COTL1, COL6A2, COL5A2, COL5A1, COL4A2, COL4A1, COL1A2, COL1A1, COL18A1, COL15A1, COL12A1, CDH11, CD74 |
| **Immune** | CCL5, CAVIN1, CALD1, BGN, AEBP1, ACTA2, A2M |
| **migration_proliferation** | VIM, TAGLN, FN1, MARCKS, SPARCL1, SPARC, CCL5 |
| **epithelial_tumor** | EPCAM, KRT18, KRT19, SNCG, POSTN |
| **smooth_muscle** | VIM, ACTA2, TAGLN |
| **macrophage** | MRC2, MFGE8, LSP1 |

**Supplementary Table2: Upregulated genes shared by the four samples.**

* ECM: extracellular matrix
