# Supplementary material for: Spatial multi-omics analysis of tumor-stroma boundary cell features for predicting breast cancer progression and therapy response
Source: Front Cell Dev Biol. 2025 Mar 26;13:1570696. doi: 10.3389/fcell.2025.1570696 (PMC11979139; doi:10.3389/fcell.2025.1570696)
Supplement: Supplementary file 4 [file DataSheet1.pdf]

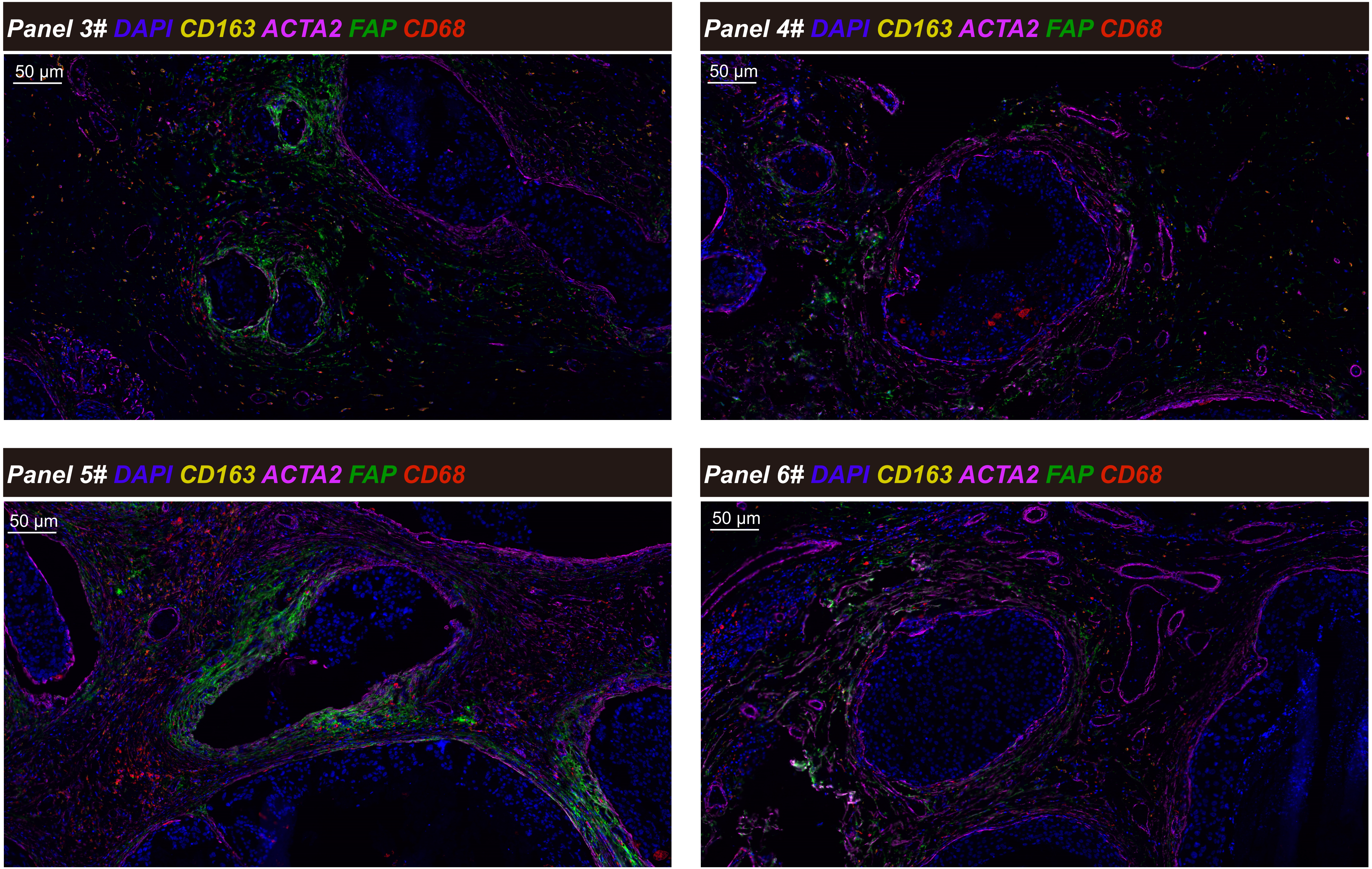

Supplementary Figure1
